# Structural Profiling of Lipid Nanoparticles at Sub‐10 nm Resolution via AF4 Coupled Online to SAXS and SANS

**DOI:** 10.1002/smtd.70639

**Published:** 2026-03-30

**Authors:** Eva Bittrich, Susanne Boye, Zanelle Van Niekerk, Zahn Stanvliet, Arthur Porfetye, Fátima Herranz‐Trillo, Hans Bolinsson, Stefaniya Gaydarova, Christo Tzachev, Anne Martel, Lars Nilsson, Ralf Schweins, Albena Lederer

**Affiliations:** ^1^ Department Advanced Macromolecular Structure Analysis Leibniz‐Institut für Polymerforschung Dresden Dresden Germany; ^2^ Department of Chemistry and Polymer Science Stellenbosch University Stellenbosch South Africa; ^3^ Wyatt Technology Europe Dernbach Germany; ^4^ CoSAXS Beamline MAX IV Laboratory Lund University Lund Sweden; ^5^ CoSAXS Beamline MAX IV Laboratory and Department of Process and Life Science Engineering Faculty of Engineering LTH Lund University Lund Sweden; ^6^ Faculty of Chemistry and Pharmacy Sofia University St. Kliment Ohridski Sofia Bulgaria; ^7^ Institut Max von Laue – Paul Langevin, DS/LSS Grenoble France

**Keywords:** AF4‐SANS, AF4‐SAXS, drug delivery system, ellipsoidal nanoparticles, lipid nanoparticles, online/in situ coupling, quinine

## Abstract

Precise mapping of structural heterogeneity at the sub‐10 nm scale is pivotal for rational nanoparticle design, yet conventional analytical workflows remain inadequate. Here we integrate dilution‐controlled asymmetric flow field‐flow fractionation (AF4) with small‐angle X‐ray scattering (SAXS) and small‐angle neutron scattering (SANS) to interrogate ellipsoidal solid–liquid lipid nanoparticles (LNPs). The dilution‐controlled AF4 mode amplifies scattering contrast, enabling robust, shape‐resolved analysis across entire elution profiles. Coupling AF4 to SANS in D_2_O further sharpens resolution for the smallest fractions by reducing particle diffusion through increased solvent viscosity. Comparative sizing shows that SAXS/SANS accurately capture primary particles down to ∼5 nm, whereas multi‐angle light scattering chiefly detects loosely associated aggregates. Morphology profiling reveals that surfactant identity governs particle shape, polydispersity, and overall architecture. Joint SAXS/SANS modeling uncovers a 2–3 nm polar shell enveloping an internal core–shell morphology. Together, these insights refine our understanding of LNP size, morphology and drug localization and establish dilution‐controlled AF4‐SAXS/SANS as a high‐resolution platform for dissecting complex nanoparticle systems relevant to biomedical applications.

## Graphical abstract

Dilution‐controlled AF4 coupled with SAXS and, for the first time, SANS enables high‐resolution characterization of non‐spherical lipid nanoparticles. Enhanced scattering contrast and D_2_O‐based measurements resolve particle features down to ∼5 nm and reveal shape distributions. Surfactant identity controls particle morphology, providing insights crucial for rational design of lipid nanocarriers.

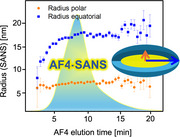

**Abbreviations**
AF4: asymmetric flow field‐flow fractionation
DLS: dynamic light scattering
FF: form factor
LNP: solid lipid nanoparticles
MALS: multi‐angle light scattering
PDI: polydispersity
SANS: small‐angle neutron scattering
SAXS: small‐angle X‐ray scattering
SEC: size exclusion chromatography
SLD: scattering length density

## 1 Introduction

Solid‐liquid lipid nanoparticles (LNPs) are part of an extended family of lipid nanoparticles and in the focus of research as drug delivery systems because of their biodegradable and biocompatible composition, improved physical stability of the rigid lipid matrix, and thus minimized drug leakage and controlled release profiles [1, 2, 3, 4]. In the present work, the term LNP is used in the broader context of lipid‐based nanoparticles, encompassing wax‐oil systems that are structurally related to solid and nanostructured lipid carriers. Structurally, LNPs are composed of rather hydrophobic solid and liquid lipids stabilized by surfactants, forming submicron particles. Their structural design enables encapsulation of both hydrophilic and lipophilic drugs, suitable for a wide range of therapeutic applications [5, 6].

Structural characterization of LNPs, in particular the determination of their shape, nanostructure, and size distributions, is a critical step in understanding their interaction with cells, in optimizing formulations, and tuning drug delivery performance [1, 4, 7]. Typically, LNPs are found to be spherical [1, 7], while a few studies show that non‐spherical shapes (platelets, ellipsoids, cylinders) are possible, dependent on the specific lipid crystallization or the processing conditions [8, 9, 10].

Size and shape determination of LNPs relies on electron microscopy in scanning (SEM) and transmission mode (TEM) [11], as well as on scattering techniques, such as dynamic light scattering (DLS) and multi‐angle light scattering (MALS) [12], laser light diffraction [12], small‐angle neutron scattering (SANS) [13], and small‐angle X‐ray scattering (SAXS) [9]. Hereby, SANS and SAXS offer the unique opportunity to highlight the internal morphology of LNPs, which cannot be achieved by probing techniques using UV or visible light.

The internal structure of lipid‐based nanoparticles is inherently complex and governed by several formulation parameters. Parameters such as the solid‐to‐liquid ratio, surfactant composition, lipid crystallinity, and processing conditions can influence particle morphology, internal structure, size distribution, and drug localization. Therefore, orthogonal high‐resolution analytical approaches are necessary to resolve structural heterogeneity in lipid‐based nanocarriers, particularly in polydisperse systems.

In principle, the accuracy of all these techniques in size determination and also in internal structure elucidation suffers from the apparent polydispersity index (PDI) of the LNPs. For drug delivery applications size and size distribution are considered as “critical quality attributes (CQAs)” by the FDA's “Guidance for Industry” on liposome drug products [14], while PDI values below 0.2 to 0.3 are generally considered acceptable for standard applications [15].

To overcome characterization uncertainties due to high PDI and the presence of impurities critical in clinical applications, fractionation techniques such as asymmetric‐flow field‐flow fractionation (AF4) have become the gold standard for precise separation and online characterization of nanoparticles by coupling with multiple detectors: MALS, UV and/or refractive index (RI) detection, and even viscometry detection [16, 17, 18, 19, 20, 21]. AF4 is particularly well suited for the separation of broad size ranges but also for delicate samples that show degradation under high shear forces or interaction typical for liquid chromatography separation. Although separation via AF4 is spanning size decades, precise size detection is limited to sizes larger than approximately 30 nm in diameter due to limitations of static light scattering, thus smaller structural features, such as the internal structure of nanoparticles, remain unresolved. Nevertheless, studies that combine fractionation by AF4 coupled online/in situ with SAXS are scarce, while reports on online/in situ AF4‐SANS are nonexistent so far. A proof‐of‐concept study of online coupling between AF4 and SAXS was performed in 2008 [22], while setup improvement and application to various biomaterial systems have been reported recently [23, 24, 25, 26].

The main challenge with this type of coupling is that, after separation, the strongly diluted fractions require considerable time to accumulate sufficient counts to produce a high‐quality signal. This time increases from MALS to SAXS and drastically to SANS, being strongly dependent on sample‐solvent contrast. Different separation approaches were therefore evaluated with variations in concentrations and flow rates in online/in situ size exclusion chromatography (SEC) coupled to SANS with limited performance [27, 28].

This study demonstrates the power of the online coupling of AF4‐SAXS and AF4‐SANS with dilution‐controlled separation for non‐spherical LNP‐formulations based on carnauba wax and red palm oil [8]. This LNP system is chosen to assess the potential of AF4 separation and subsequent SAXS and SANS detection, as well as to highlight the gain in structural information available by the optimization of separation and the combination of scattering techniques. To the best of our knowledge, this work demonstrates for the first time the online coupling of AF4 and SANS, thus offering a unique insight into distributions of sub 10 nm‐structural features.

## 2 Results and Discussion

### 2.1 LNPs

With respect to previous studies on non‐spherical LNP based on carnauba wax [8], the LNP systems depicted in Table 1 were chosen for further structural investigations by the coupling of AF4 to SAXS and SANS for online analysis.

**TABLE 1:** LNP composition series with variation in lipid, drug, and surfactant components and their diameter and PDI obtained by DLS batch measurements [8].

Sample	Lipids	Surfactants	Drug	Size (nm)	PDI
LNP‐Q	carnauba wax, red palm oil	Tween + TPGS	quinine	35.7 ± 0.3	0.151 ± 0.017
LNP‐p	carnauba wax, red palm oil	Tween + TPGS		38.1 ± 0.1	0.126 ± 0.006
LNP‐CW‐Tween	carnauba wax	Tween		48.5 ± 0.1	0.164 ± 0.006
LNP‐CW‐TPGS	carnauba wax	TPGS		35.4 ± 0.4	0.175 ± 0.011

Hereby, LNP‐p resembles the placebo particle and LNP‐Q the quinine loaded one, both based on LNPs patented as CellInject for drug delivery applications [29]. Variation of the surfactant composition in LNP‐CW‐Tween and LNP‐CW‐TPGS further elucidates the influence of Polysorbate 40 (Tween) and vitamin E‐based d‐α‐tocopherol‐polyethylenglycol‐1000‐succinate (TPGS) on the particle morphology. These LNP consist solely of the lipid carnauba wax and the respective surfactant.

### 2.2 Enhanced AF4‐SAXS Resolution

**FIGURE 1:**
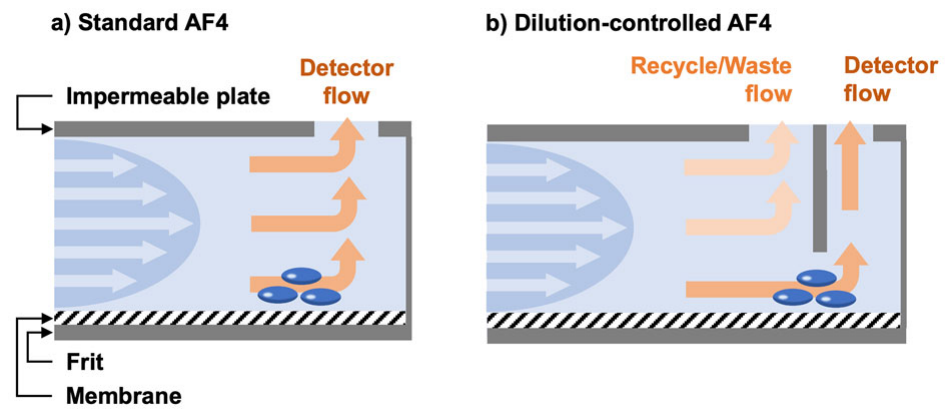
Cross‐section of different channel designs: a) standard AF4 and b) dilution‐controlled AF4 for the separation of nanoparticles.

Key limitations of conventional AF4 in coupling with SAXS are the restricted injection volume due to channel overloading and the sample dilution during separation. These constraints significantly limit the sample concentration, thereby reducing SAXS signal intensity. To address excessive dilution during AF4‐SAXS coupling, we implemented an AF4 mode incorporating a dedicated Dilution Control Module (DCM). The DCM enables a controlled flow split directly at the channel outlet, i.e., after the size separation is complete. Under conventional AF4 operation, the entire cross‐section of the channel outlet, including both the particle‐rich lower region and the solvent‐rich upper region, is reaching the detectors, resulting in substantial dilution, particularly for low‐concentration samples. In the DCM mode, the outlet stream is divided into two adjustable fractions. The lower, analyte‐rich layer of the parabolic channel flow profile is directed to the detectors, whereas the upper, mostly particle‐free layer is removed as waste. This selective removal of the solvent‐rich portion preserves the peak concentration and delivers a stable stream to downstream detectors, leading to enhanced SAXS signal intensity without altering separation conditions inside the AF4 channel (Figure 1, details on the DCM approach can be found in Section S1).

**FIGURE 2:**
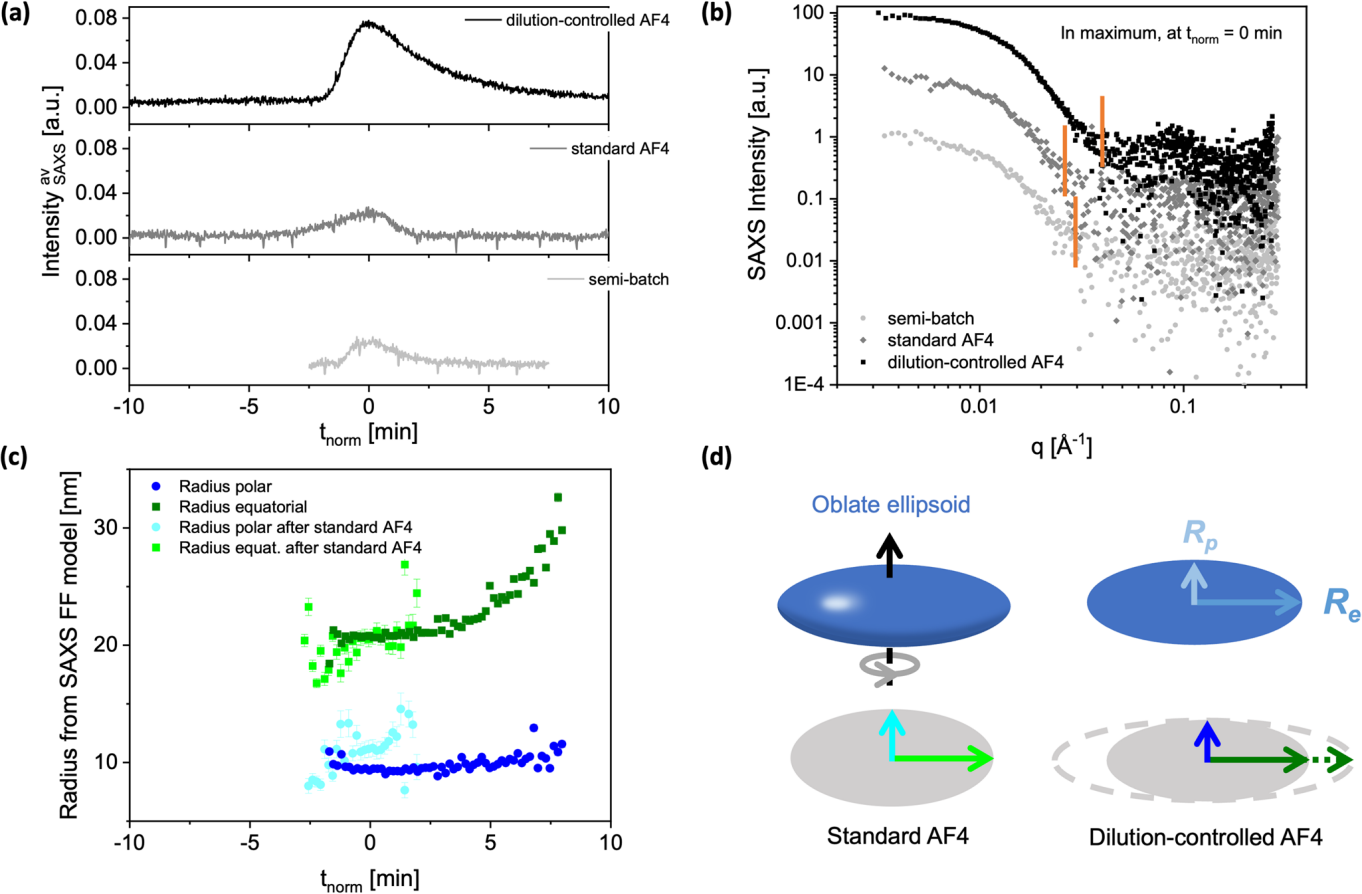
Comparison of SAXS data for quinine‐loaded LNPs (LNP‐Q) following different separation modes: (a) Time‐resolved SAXS intensity averaged across the full q‐range, normalized to the time‐point of maximum intensity to allow comparison between different separation modes (for data with absolute time scale see SI, Figure S13), (b) SAXS data at the elution maximum, with the high q‐limit indicated for evaluation, (c) Comparison of polar (*R_p_*) and equatorial (*R_e_*) radii of the elliptical LNPs obtained from form factor (FF) modeling of SAXS data after standard and dilution‐controlled AF4 separation, and (d) Schematic illustrations of the oblate ellipsoid, definitions of *R_p_* and *R_e_*, and the interpretation of SAXS‐derived shape differences at the elution maximum in the two AF4 modes, also indicating the progression in *R_e_* after dilution controlled AF4.

As shown in Figure 2a for the quinine‐loaded LNPs, dilution‐controlled AF4 results in substantially higher average SAXS intensities compared to standard AF4 and measurements in semi‐batch mode (flow without separation), enabling more sensitive and precise structural characterization. The improved SAXS statistics extend the accessible scattering vector (q) range (Figure 2b), which is critical for the accurate determination of particle shape. SAXS data in the elution maximum with signal‐to‐noise ratio (SNR) > 2 can be obtained up to q‐values of 0.04 Å^−1^ as shown for LNP‐Q which corresponds to length scales of approx. 15 nm. For SAXS data in semi‐batch mode and using standard AF4 q‐limits for similar SNR would be only 0.03 Å^−1^ (20 nm) and 0.025 Å^−1^ (25 nm) respectively.

Notably, the broader SAXS peak after dilution‐controlled AF4 results from a lower detector flow of 0.2 mL/min as compared to 0.5 mL/min for standard AF4. For a comprehensive description of AF4 separation approaches and data see also Supporting Information (AF4 systems: Figures S1 and S2, AF4 data: Figures S5 and S6, SAXS data: Figure S13).

The higher SAXS quality after dilution‐controlled AF4 enhances the contrast along the elution profile. This increased contrast also expands the range of the fractogram for which high‐resolution SAXS data are obtained (Figure 2c), thereby allowing for more comprehensive structural analysis over a larger portion of the sample population. This improvement is particularly evident in the derived shape parameters of the investigated LNP‐Q. The dimensions of separated LNPs were calculated using a form factor model of an ellipsoid of revolution with rotational symmetry around the polar (short) axis [30]. Figure 2c compares the polar (*R_p_*) and equatorial (*R_e_*) radii extracted from SAXS. Clearly, a standard AF4 limits the range of fractions which can be interpreted via online SAXS. In contrast, SAXS data collected after dilution‐controlled AF4 reveal smaller *R_p_* for a wide range of elution times, indicating a pronounced elliptical shape (Figure 2d). On the one hand, the optimized separation increases the quality of the SAXS data, leading to higher resolution plateau‐values for polar and equatorial radius ($R_{p}^{SAXS}=\left(9.3\pm 0.1\right)$ nm and $R_{e}^{SAXS}=\left(20.8\pm 0.2\right)$ nm). On the other hand, the expanded SAXS‐fractogram unveils a distinct size progression for *R_e_* from 20.4 to 29.8 nm vs almost constant *R_p_*—a significant distribution in particle shape, not visible in batch SAXS or standard AF4‐SAXS.

In addition, dilution‐controlled AF4 allows an insight into a broad peak at 0.09 Å^−1^ corresponding to a length of ≈ 7 nm (Figure 2b), which is detected for all LNPs (see Figure S17a). Briefly, the peak could either stem from a surfactant‐bilayer structure factor [9] or the lamellar stacking of crystalline wax esters within the carnauba wax [31]. The internal structure of the particle is discussed more closely in our previous report [8]. In the current study, we focus on the overall shape and size distribution of the LNPs.

In the same previous work [8], we observed deviations between the LNPs aspect ratios (*R_e_*: *R_p_*) obtained from orthogonal characterization via batch laboratory SAXS (2:1) and cryo‐TEM experiments (4:1). It is obvious that batch lab SAXS delivers average values (in addition to lower resolution compared to synchrotron SAXS), while cryo‐TEM gives only discrete values of a very limited number of particles. Thus, separation prior to scattering detection is the only reliable method to get the full distribution picture: an aspect ratio of 2:1 during the first 4 min of the separation, which increases to approximately 4:1 during subsequent elution until complete separation (Figure 2c).

### 2.3 Resolving sub‐7 nm‐Distributions via AF4‐SANS

**FIGURE 3:**
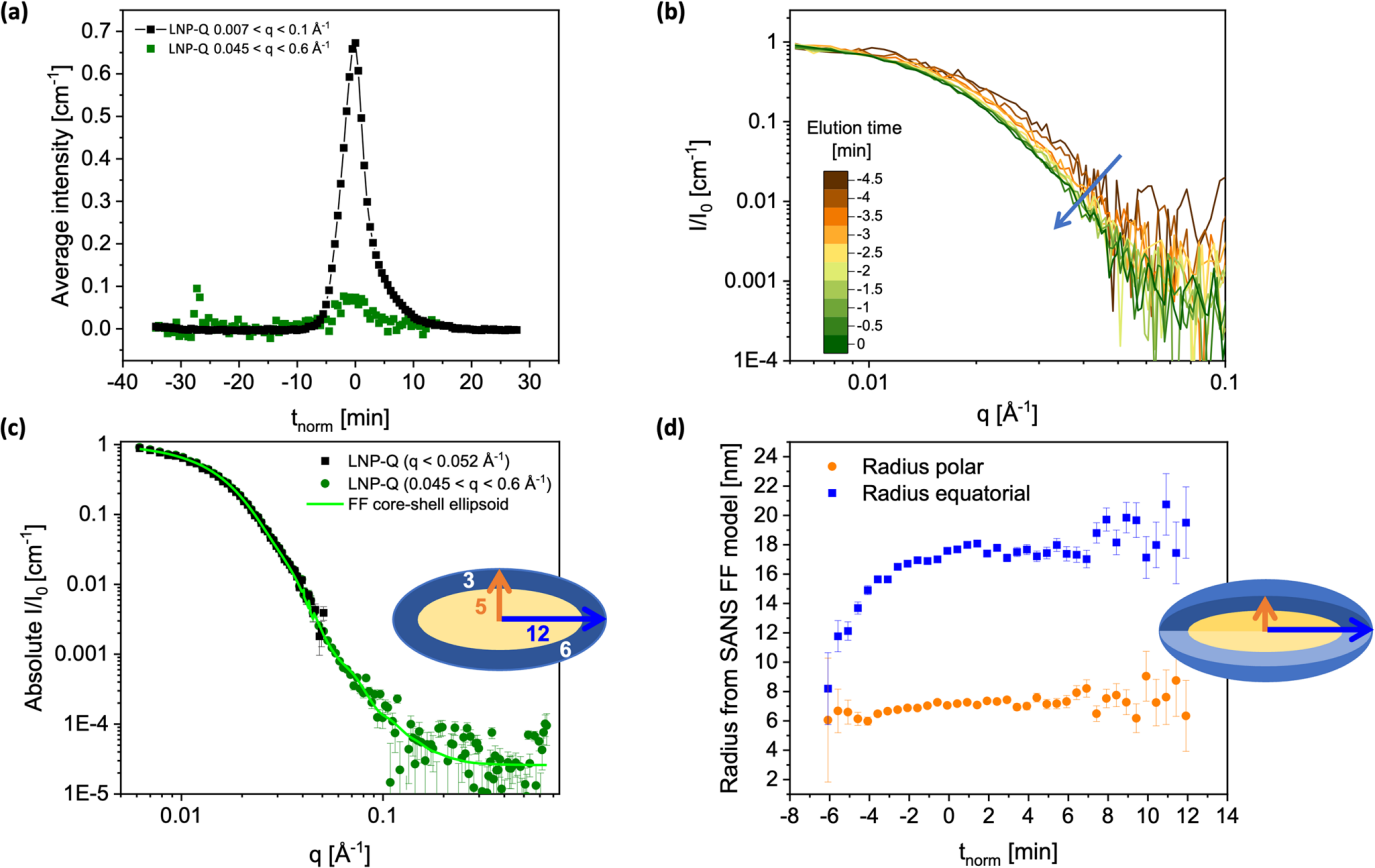
SANS data for quinine‐loaded LNP (LNP‐Q) after dilution‐controlled AF4: (a) averaged intensity vs. time for two detector distances (low q‐range at 10.2 m and high q‐range at 1.6 m; for data with absolute time scale see Figure S21), (b) individual SANS curves for 30s frames collected between 30–35 min elution time at 10.2 m detector distance, (c) fit of core–shell ellipsoid FF model to the SANS data at 35 min (low q‐range), combined with the high q‐range (green circles from (a), integrated over the elution peak due to low intensity), and (d) *R_p_* (orange) and *R_e_* (blue) obtained from the ellipsoid FF model as a function of elution time. All dimensions shown in the schematics are in [nm].

Compared to SAXS, SANS offers a variation of contrast which has the potential to increase the information density of an AF4‐online experiment. A first‐of‐its‐kind online dilution‐controlled AF4‐SANS experiment was thus performed on the same LNPs analyzed with AF4‐SAXS. We obtained a sufficient SANS contrast in the range 0.007 < q < 0.1 Å^−1^ (90> d > 6 nm) as shown for LNP‐Q (Figure 3a; Figure S21a), which significantly extends to higher q as compared to SAXS, allowing deeper insight into the LNP structure. The scattering intensities vs. q vary in the first 5 min of the separation (Figure 3b), while I(q) remains constant beyond the elution maximum (Figure S21b). This is opposite to the observed trends in AF4‐SAXS with constant I(q) before the elution maximum and changes in the SAXS scattering profile at a later elution time (Figure S14).

At the elution maximum, the low q‐range SANS data (0.007 < q < 0.1 Å^−1^) was combined with the integrated data of the high‐q‐range (0.045 < q < 0.6 Å^−1^). The integration over the complete peak was required due to insufficient intensity of the single data point (Figure 3a, green fractogram). The resulting scattering curve was modelled with a core–shell ellipsoid FF (Figure 3c; Figure S24a). The overall dimensions derived from the FF model at the maximum were $R_{p}^{SANS}=\left(7.7\pm 0.4\right)\;$ nm, and $R_{e}^{SANS}=\left(18.2\pm 0.9\right)\;$ nm aligning well with AF4‐SAXS‐derived sizes (Figure 2c). Normalization of the SANS data, i.e. determining the intensity I_0_ (Figures S22 and S23), allows to evaluate the scattering length densities of the core and the shell. For LNP‐Q, we find a core with slightly lower scattering length density $SLD_{core}^{SANS}=\left(6.351\pm 0.001\right)\;$ 10^−6^/ Å^2^ as compared to the shell $SLD_{shell}^{SANS}=\left(6.359\pm 0.001\right)\;$ 10^−6^/ Å^2^. Although the data is not corrected for the (unknown) absolute particle concentration, these values allow for a relative comparison and confirm that the shell and core of the LNP exhibit different contrasts in D_2_O. Based on the theoretical SLDs of the LNP components (Table S5), one interpretation of the data could be a particle internal structure of a more solid core and a more swollen shell in D_2_O. However, previous SAXS and cryo‐TEM studies suggest rather incorporated water in the core [8]. This is a more plausible explanation of the SLD contrast between core and shell, considering (i) a proton‐rich core, due to encapsulated H_2_O from the particle formation step and (ii) the fact that the hydrophobic shell is most probably not penetrable for D_2_O. Considering that the non‐loaded LNP‐p has a similar core–shell structure (Figure S24c), we calculate the same core SLD but higher $SLD_{shell}^{SANS}=\left(6.365\pm 0.001\right)\;$ 10^−6^/ Å^2^. The composition of LNP‐Q and LNP‐p differs only in the quinine content. This fact and the lower SLD of LNP‐Q in the shell is a clear indication, that the quinine (SLD in Table S5) is preferentially located in the shell rather than in the core.

Focusing again on the size evolution from the SANS fractogram collected with sufficient SNR at 0.007 < q < 0.1 Å^−1^, FF modeling of an ellipsoid of revolution was successfully applied. Hereby, a different trend compared to AF4‐SAXS was observed (Figure 3d vs. Figure 2c): The equatorial radius modeled from SANS increased during early elution before reaching the plateau ($R_{e}^{SANS}$∼18 nm), while constant values of $R_{e}^{SAXS}$∼20.7 nm from SAXS were obtained before the elution maximum. The polar radius remained constant during the separation for both SANS and SAXS, but with ∼2‐3 nm smaller SANS‐values.

The general discrepancy between SANS and SAXS derived sizes, with SANS sizes being systematically smaller than those from SAXS, may stem from screening of the outer polar layer by deuteration in D_2_O, potentially reducing the apparent shell thickness in the SANS measurements.

Comparing the time progression of average detected intensities after dilution‐controlled AF4 from SAXS (Figure 2a, UV‐signal in Figure S6) and SANS (Figure 3a, UV‐signal in Figure S7), it is evident that the main elution peak in the SAXS experiment was considerably narrower than for SANS. An obvious reason for this is that the AF4‐SANS separation takes place in D_2_O, which possesses higher viscosity compared to H_2_O, which influences the separation efficiency. In addition, SAXS and SANS allow for detection in different size ranges, with SANS resolving a broader size range (Figure 3). To understand these effects, a precise comparison to other detectors coupled within the AF4 multidetector system is considered in the next section.

### 2.4 Which is the Correct Dimension?

To critically assess the accuracy of particle size determination, we compared radii obtained from light scattering (MALS, DLS) and those derived from SAXS and SANS following dilution‐controlled AF4. For this purpose, we focus on the non‐loaded, placebo LNP‐p.

**FIGURE 4:**
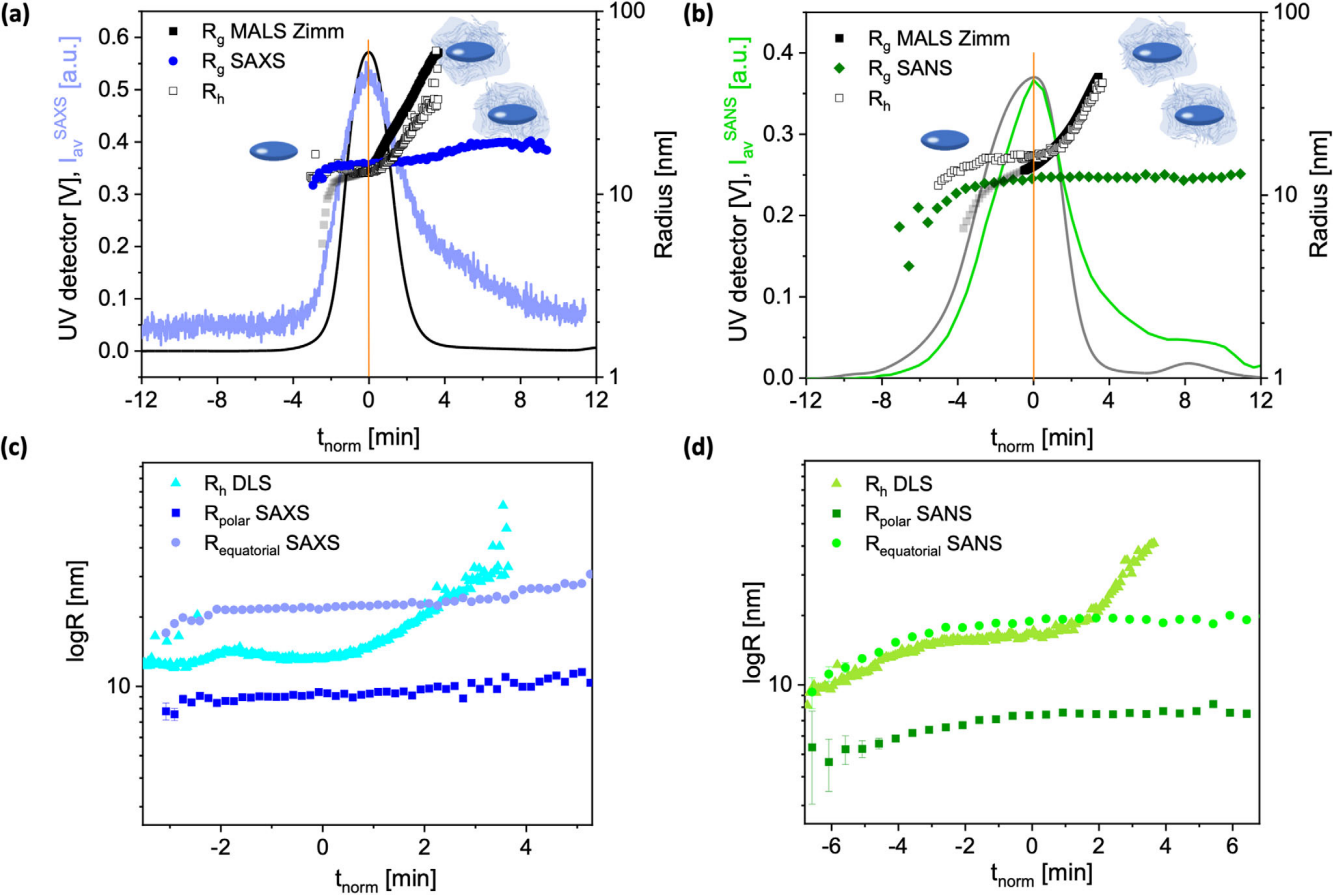
Comparison of evaluated sizes derived from light scattering (MALS, DLS) and SAXS (a,c) as well as from MALS, DLS (after separation in D_2_O), and SANS (b, d) for the placebo LNP‐p. (a,b) Concentration‐sensitive UV signal, average intensity, hydrodynamic radius (*R_h_*) from online DLS and radius of gyration (*R_g_*) as derived from Zimm plots (MALS) and Guinier analysis (SAXS/SANS). (c,d). Form factor (FF) model parameters (*R_p_* and *R_e_*) of ellipsoidal fits, compared to DLS‐derived *R_h_*.

For better comparison, the zero‐point of time axes of concentration‐dependent UV signals and scattering intensities (SAXS, Figure 4a; SANS, Figure 4b) was shifted to the elution peak maximum. Notably, SAXS and SANS exhibited signal broadening as compared to the UV signal, attributed mainly to a large inter‐detector volume after AF4. In both cases, band broadening at high elution times occurs due to the low flow rate of 0.2 mL/min. *R_g_* values from SAXS and SANS (Figure 4a,b) remained relatively constant, though SANS‐derived *R_g_* was systematically lower—consistent with prior LNP‐Q model data. Remarkably, in the elution maximum the same *R_g_* of 14.4 ± 0.6 nm is obtained from SAXS and MALS data. This value also corresponds to the lower theoretical limit of static light scattering (∼16 nm).

Before the elution maximum, *R_g_* from SAXS and SANS are higher than MALS‐derived *R_g_*. Figure S9 indicates that the Zimm plot fit quality is below 90% up to the maximum of the elution curve, which confirms that static light scattering (λ = 660 nm) is limited for sizes below λ/20. In contrast, SAXS and SANS deliver reliable sizes in this range.

The increase in MALS‐derived *R_g_* after the elution maximum cannot be found in the data from SAXS and SANS. MALS probes close to zero angle and is therefore highly sensitive to a small fraction of larger species (> 20 nm). In contrast, our SAXS and SANS (q > 0.003 and 0.007 Å^−1^, respectively) data only partly cover, or completely miss, the Guinier regime of these aggregates, so that they contribute only a weak low‐q signal. At the late elution times where MALS detects these species, their volume fraction is low and the primary LNPs (< 20 nm) dominate the SAXS/SANS signal, placing any aggregate contribution within the noise level. This difference rather indicates contributions from loosely bound aggregates/associates with diluted interconnected shells, detectable by MALS, but not by SAXS/SANS. The relatively high concentrations used to obtain reliable SNRs (1 mg/mL (SAXS) and 2.7 mg/mL (SANS)) could promote aggregate formation, which in fact, was not observed at lower concentrations relevant in drug delivery systems [8]. Given the low scattering contrast between the interconnecting shell and the eluent in SAXS and SANS, these techniques primarily resolve the individual particles. Co‐elution of differently sized species can be ruled out, as evidenced by the consistent fits of the DLS correlation curves across the fractograms, which confirm narrow size‐distributed fractions (Figures S10 and S11). In summary, MALS reliably maps the size distribution beyond the elution maximum (for values larger than approximately λ/20), whereas SAXS and SANS provide precise measurements of the individual LNP dimensions. This interpretation is further supported by the distribution of *R_h_* across the entire peak obtained by online DLS, which, unlike MALS, does not depend on anisotropic scattering but rather on fluctuations in scattering intensity. The *R_h_* trend aligns well with the above conclusions. However, DLS remains limited to probing the global hydrodynamic size associated with diffusion properties and does not provide structural information.

To shed light on the LNP structure, next to *R_g_* determination, FF modeling was performed for the LNP‐p and discussed in relation to *R_h_* (Figure 4c,d). If we compare equatorial and polar radii from SAXS and SANS again, systematically smaller radii from SANS are evident. As for the LNP‐Q particles, $R_{e}^{SANS}$ increases up to the elution maximum, while $R_{e}^{SAXS}$ is constant before reaching the maximum. Since these findings for LNP‐p and LNP‐Q could, in principle, also stem from composition‐dependent deuteration effects, we now have a closer look at the hydrodynamic radius derived from DLS data. The detected hydrodynamic radii differ slightly between AF4‐DLS‐SAXS and AF4‐DLS‐SANS, but follow the trends observed in SAXS and SANS at lower elution times (Figure 4c,d). *R_h_* follows remarkably well the trend observed for $R_{e}^{SANS}$ up to the maximum of the elution curve, which as a hydrodynamics‐based value matches the expectations for ellipsoidal particles. Beyond the maximum, *R_h_* again indicates larger particle sizes, while sizes derived from SANS stay constant. Hereby, the same trends in *R_h_* and $R_{e}^{SANS}$ before the elution maximum confirm improved separation efficiency of AF4 in D_2_O compared to H_2_O. We propose that the experiments conducted in D_2_O provide improved resolution in separating smaller particles compared with those performed in a standard water‐based buffer. The higher viscosity of D_2_O reduces the diffusion of all particles. Combined with the high sensitivity of SANS in the lower size range, this enables the detection of the smallest particle populations.

In summary, particle separation and size detection by different scattering detectors lead to complementary insights into size distributions, elucidating particle shape and composition progression in a comprehensive manner.

### 2.5 The Role of the Surfactants in LNP Formation

Surfactants play a decisive role in self‐assembly, size distribution, and shape of LNPs. In a previous report, we found that the dual‐surfactant system comprising TPGS and Tween yields LNPs with narrow size distributions and low polydispersity. In contrast, single‐surfactant systems (TPGS or Tween alone) produce broader size distributions, indicative of less controlled assembly. The stabilization provided by both surfactants was sufficient to prevent aggregation [8].

**FIGURE 5:**
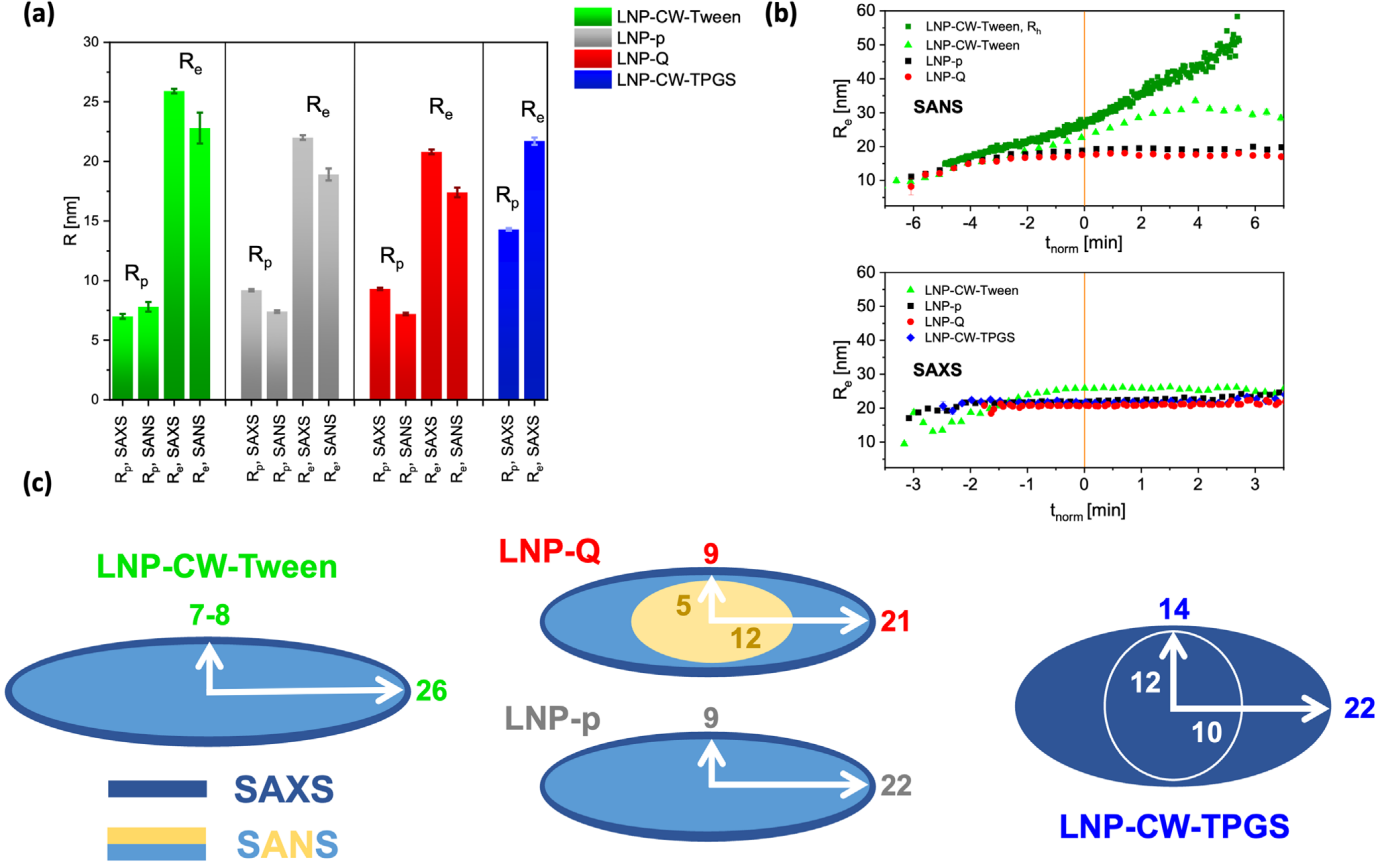
(a) Size parameters (*R_p_* and *R_e_*) derived from form factor modeling of SAXS and SANS data for LNP‐CW‐Tween (green), LNP‐p (grey), LNP‐Q (red), and LNP‐CW‐TPGS (blue). Values represent averages over a 1‐minute interval at the elution maximum. *Note*: SANS data for LNP‐CW‐TPGS is not available. (b) *R_e_* as a function of elution time for the LNP systems, derived from SANS and SAXS. The hydrodynamic radius (*R_h_*, dark green) from DLS is shown for LNP‐CW‐Tween, as obtained from the AF4‐SANS setup. (c) Schematic illustrations of particle sizes in [nm], geometry, and internal interfaces, modeled from SANS or SAXS data at the elution maximum for all LNP systems.

To probe the effect of the type of surfactant on particle geometry, we evaluated LNPs composed solely of carnauba wax and stabilized by either Tween 40 (LNP‐CW‐Tween) or TPGS (LNP‐CW‐TPGS), alongside dual‐surfactant and drug‐loaded systems (LNP‐p and LNP‐Q). SAXS‐ and SANS‐derived ellipsoidal parameters at the maximum of the elution curve revealed a clear surfactant‐dependent trend (Figure 5a): the polar radius (*R_p_*) increased from LNP‐CW‐Tween < LNP‐Q ≈ LNP‐p < LNP‐CW‐TPGS, while the *R_e_* was highest for LNP‐CW‐Tween, and lower but similar for all other particles containing TPGS (Figure 5a). These trends indicate that Tween 40 favors more elongated, elliptical particles, whereas TPGS promotes less elongated shapes, an important consideration for drug delivery applications where particle shape can influence biodistribution and cellular uptake [32]. Notably, all carnauba‐wax‐only formulations still displayed ellipsoidal geometries (*R_e_*/*R_p_*  =  1.6 for LNP‐CW‐TPGS), highlighting the intrinsic tendency of carnauba wax to form non‐spherical particles. For the dual‐surfactant formulations loaded with (LNP‐Q) or without drug (LNP‐p), the aspect ratio *R_e_*/*R_p_* was 2.2 to 2.5. The anisotropic shape of all particles is also confirmed by the shape parameter calculated from the ratio *R_g_/R_h_* in the range 0.65–1.3 (Figure S12 and Table S4).

As discussed before, the *R_e_* values derived from SANS were systematically smaller (3.2 ± 0.2 nm) than *R_e_* derived from SAXS. For LNP‐p and LNP‐Q, also the *R_p_* from SANS were smaller (2.0 ± 0.2 nm). Since both Tween 40 and TPGS include PEG segments (on average 20–22 units), we infer that the outer shell is formed by the surfactant headgroups, corresponding to approx. 3.4 nm thickness as indicated in previous SAXS studies [9]. Notably, LNP‐CW‐Tween showed broad *R_e_* (Figure 5b) and *R_p_* distributions as compared to the narrow distribution of *R_p_* for TPGS containing particles (Figures S18 and S25), indicating a polydisperse mixture of elliptical particles. Here, the *R_h_* correlates well with *R_e_* from SANS, suggesting a genuine size progression of the elliptical LNP‐CW‐Tween particle shape, with an aspect ratio of *R_e_*/*R_p_*  =  3.3 ± 0.4 in the maximum of the elution curve (Figure 5a).

For LNP‐CW‐TPGS, a core could be modelled from SAXS data in the elution maximum utilizing a core–shell ellipsoidal FF (sketch Figure 5c; Figure S17). The resolved core has an almost spherical shape with a radius of 10–12 nm, comparable to the extended length of the LNP‐Q and LNP‐p cores (Figures 3c and 5c). Since LNP‐CW‐TPGS consists solely of carnauba wax and TPGS, this implies that the core–shell structure is defined by these two components in the LNP. In contrast, a core–shell structure could not be found for the LNP‐CW‐Tween system (Tween 40 + carnauba wax, SAXS data Figure S17a, SANS data Figure S24b). SAXS intensity normalization enabled fitting of the SLDs for these two LNPs: For the LNP‐CW‐Tween particle, without core–shell characteristics, we obtain an *SLD^SAXS^* =  9.419 × 10^−6^/Å^2^, which is an average of the SLD values for the core and the shell in LNP‐CW‐TPGS ($SLD_{core}^{SAXS}=\left(9.428\pm 0.001\right)\times 10^{-6}/{Å}^{2}\;$ and $SLD_{shell}^{SAXS}=\left(9.409\pm 0.001\right)\;\times 10^{-6}/{Å}^{2}$). Compared to theoretical SLDs (Table S5), this result suggests a diluted core and a more compact shell for LNP‐CW‐TPGS, which is in contrast to reported LNP structures [32] but in agreement with our conclusions made from the SANS analysis of LNP‐Q and LNP‐p and confirmed by batch lab SAXS analysis combined with cryo‐TEM [8].

## 3 Conclusion

AF4 coupled online to SAXS—and crucially, for the first time to SANS—has been established as a powerful platform for interrogating non‐spherical lipid nanoparticles (LNPs) composed of carnauba wax, red palm oil, and the PEG‐based surfactants Tween 40 or TPGS. Introducing dilution‐controlled AF4 markedly enhanced SAXS/SANS contrast, allowing form factor analysis up to q ≈ 0.1 Å^−^^1^ and enabling clear, elution‐resolved discrimination between equatorial and polar particle dimensions.

Operating the AF4‐SANS set‐up in D_2_O extended reliable structural assessment across q = 0.007–0.1 Å^−^^1^. Particle radii determined by SANS were systematically smaller than those from SAXS, consistent with deuteration of the outer polar shell. The higher viscosity of D_2_O also sharpened the separation of the smallest fractions and enabled the profiling of a core–shell LNP shape in which the loaded drug is clearly located in the shell region.

While multi‐angle light scattering (MALS) underestimated radii below ∼14 nm, it uniquely revealed loosely associated aggregates that remained undetected by both SAXS and SANS.

Surfactant choice proved to be a decisive factor in determining particle morphology: Tween 40‐stabilised LNPs exhibited equatorial radii with pronounced polydispersity, whereas TPGS‐stabilized LNPs were more spherical and monodisperse. SAXS modeling of the TPGS system uncovered a core–shell architecture with a dense, carnauba‐wax‐rich shell.

Collectively, these results provide a detailed picture of LNP shape evolution, surfactant effects, and elution‐dependent size distributions. Beyond the specific systems studied, the work demonstrates dilution‐controlled AF4‐SAXS/SANS as a robust, high‐resolution strategy for nanoparticle characterization, opening avenues for deeper understanding and ultimately rational design of nanocarriers for biomedical applications.

## 4 Experimental Section/Methods

### 4.1 LNPs

LNPs, patented as CellInject, were prepared as described elsewhere [8, 29]. Briefly, carnauba wax (1.00–5.00 w/w parts), red palm oil concentrate 0.10–0.50 w/w parts), d‐α‐tocopheryl polyethylene glycol 1000 succinate (TPGS) (0.70–3.50 w/w parts) and Polysorbate 40 (Tween) (0.50–2.50 w/w parts) are mixed and heated up to 90°C under stirring until a clear melt mixture is obtained. Quinine (0.1–0.5 w/w parts) is added to the mixture at 90°C for LNP‐Q. Pre‐heated NaCl (0.9 w/w parts) in deionized water (90°C) is added dropwise to the mixture. Finally, the dispersion is cooled down to 20°C under stirring. For LNP‐CW‐Tween and LNP‐CW‐TPGS only carnauba wax and Tween 40/TPGS are used respectively in the initial mixture.

### 4.2 Asymmetrical Flow Field Flow Fractionation (AF4)

All AF4 experiments were performed using an identical channel configuration comprising a trapezoidal short channel (tip‐to‐tip length 175 mm, maximum width 21 mm) with a 350 µm spacer thickness, fitted with a 10 kDa regenerated cellulose (RC) membrane. The specific AF4 systems used are detailed below. A detailed description of the Dilution Control Module (DCM) is given in the Supporting Information.

### 4.3 Standard AF4 (System 1)

AF4 separations were conducted using an Eclipse 3+ field‐flow fractionation system (Wyatt Technology, Santa Barbara, CA, USA. The separation system was coupled to the following detectors: A DAWN EOS MALS detector (Wyatt Technology, λ = 660 nm) and a diode array UV–vis detector 1260 (Agilent Technologies, USA, λ = 280 nm). The eluent consisted of ultrapure water. Semi‐batch measurements were performed by direct channel injection without applied focus and cross‐flow at a flow rate of 0.5 mL/min. A total of 0.260 mg of sample (1.04 mg/mL) was loaded by injecting 250 µL. See Figure S1 for the optimized separation profile.

### 4.4 Dilution‐Controlled AF4 (Systems 2 and 3)

AF4 separation was performed using an Eclipse NEON system (Waters | Wyatt Technology, USA) with Dilution Control Module (DCM) option coupled to a multi‐detector setup consisting of a (1) DAWN NEON MALS detector (Waters | Wyatt Technology, USA, λ = 660 nm) with DLS option, (2) an Optilab T‐rEX differential refractive index (dRI) detector (Waters | Wyatt Technology, USA, λ = 660 nm), and (3) UV detector 1260 (Agilent Technologies, USA) (system 2)/UV detector SPD‐30 (Shimadzu, Japan) (system 3). The integrated Dilution Control Module (DCM) minimizes sample dilution without compromising resolution, improving fraction concentration and sensitivity. The split ratio (SR) of DCM flow and detector flow was set to 8.

For system 2, a total of 0.260 mg of the sample (1.04 mg/mL) was loaded by injecting 250 µL. For system 3, the MALS detector was equipped with wide‐bore flow cells, attenuators, and fluorescence filters. The SANS flow cell was placed in between the MALS and the dRI detector. The eluent consisted of 99.9% D_2_O. A total of 0.405 mg of sample (2.7 mg/mL) was loaded by injecting 150 µL. For more details on systems 2 and 3, and for the optimized separation profiles see the (Figures S2 and S3).

### 4.5 MALS

Astra 8.3 software (Waters | Wyatt Technology, USA) was used for the calculation of size distributions. The angle‐dependent scattering of the LNP systems was evaluated using the Zimm model which was found suitable in the regime of small particle radii (below 50 nm). It provides markedly higher fitting stability and often improved robustness compared to Berry or Debye fits. For LNPs, the angular dependence is weak, and the linear Zimm approximation is less sensitive to noise and less prone to overfitting in low singal to noise regimes than higher‐order Berry/Debye models.

### 4.6 SAXS

Measurements were performed at the CoSAXS beamline at MAX IV (Lund, Sweden) at a wavelength of 0.99 Å. The data was collected on an Eiger2 4 M (Dectris) detector within an evacuated flight tube, positioned at 3.5 m distance (0.003 < q < 0.29 Å^−1^), and at a scan rate of 1 s per frame. The flow cell consists of a 1.5 mm inner‐diameter quartz capillary, with a 10 µm wall thickness. The sample in 1 mm PBS buffer passed the SAXS cell at a flow rate of 0.2 mL/min after dilution‐controlled AF4. Reduction of the 2D scattering pattern to 1D scattering intensity was performed and the data corrected for the empty buffer background (integration of 300 frames), subsequently. 2D data was controlled for anisotropy (Figure S20). 1D SAXS data at low q was analyzed by Guinier fits, and *R_g_* and *I*_0_ extracted framewise by self‐written R‐scripts. The SAXS data was normalized to *I*_0_ to correct for concentration dependency. Normalized data was fitted framewise to FF models of ellipsoid or core–shell ellipsoid of revolution (about the short axis) using SasView [30]. A cylindrical FF was also tested, which led to similar sizes (LNP‐Q), but had lower fit quality.

### 4.7 SANS

Small angle neutron scattering measurements [33] were carried out at the D11 instrument of the ILL (Grenoble, France), at a neutron wavelength of 5.24 Å (chosen for the highest available flux). Integration time was 30 s per frame. The sample‐to‐detector/collimation distances were at 10.2 m/10.2 m (0.007 < q < 0.1 Å^−1^), and also at 1.6 m/4.1 m (0.045 < q < 0.6 Å^−1^) for selected samples. A home‐built SANS flow‐through cell [28] was used with an exposed volume of 133 µL (11 mm diameter and 1 mm thickness). The sample in 99.9% D_2_O (1 mm PBS) buffer passed the SANS cell at a flow rate of 0.2 mL/min after dilution‐controlled AF4. 2D SANS data was checked for anisotropy (Figure S27), scaled to absolute intensity using direct flux measurement and transmission‐corrected. As a background, the cell and buffer contributions were subtracted as well as the blocked beam contribution. Patterns were reduced to 1D scattering curves using the software GRASP [34]. For Figure 3a, intensity per pixel was averaged from 2D data over 2 circular boxes: one from q = 0.007 to q = 0.1 Å^−1^, and one from q = 0.045 to q = 0.6 Å^−1^. 1D SANS data at low q was analyzed by Guinier fits, and *R_g_* and *I*_0_ extracted framewise by self‐written R‐scripts. The SANS data was normalized to *I*_0_ to correct for concentration dependency. Normalized data for 0.007 < q < 0.1 Å^−1^ was fitted framewise to the FF of an ellipsoid of revolution (about the short axis) using SasView [30]. In the maximum of evolution, data for LNP‐Q and LNP‐p was stitched to the integrated data of 0.045 < q < 0.6 Å^−1^ using PRIMUS [35], and fitted using a core–shell ellipsoid FF model using SasView [30].

All reported errors for the SAXS and SANS data correspond to one standard deviation of the respective fitted parameter.

## Author Contributions

The manuscript was written through the contributions of all authors. All authors have given approval to the final version of the manuscript. S. Boye and E. Bittrich made equal contributions to this work.

## Funding

A. Lederer and Z. Van Niekerk acknowledge funding within the 3D4D2 project carried out under the M‐ERA.NET scheme (European Union's Horizon 2020 research and innovation programme, grant No. 685451) and co‐funded by the Saxon State Ministry for Science, Culture and Tourism (Germany), grant No. 100579959, as well as from the tax funds based on the budget passed by the Saxon state parliament. LN acknowledges funding from the Swedish Research Council (VR)grant “2021‐04667_VR”, the Crafoord foundation, Lund, Sweden, and The Royal Physiographic Society of Lund, Sweden. Funding was received for the experiments at Institut Laue‐Langevin (https://doi.org/10.5291/ILLDATA.9‐13‐1177 and https://doi.org/10.5291/ILL‐DATA.9‐13‐1178).

## Conflicts of Interest

The authors declare no conflicts of interest.

## Supporting information




**Supporting File**: smtd70639‐sup‐0001‐SuppMat.pdf.

## Data Availability

The data that support the findings of this study are available on request from the corresponding author. The data are not publicly available due to privacy or ethical restrictions.
