# Supplementary material for: Structural Profiling of Lipid Nanoparticles at Sub‐10 nm Resolution via AF4 Coupled Online to SAXS and SANS
Source: Small Methods. 2026 Mar 30;10(12):e70639. doi: 10.1002/smtd.70639 (PMC13288020; doi:10.1002/smtd.70639)
Supplement: Supplementary file 1 — Supporting File: smtd70639‐sup‐0001‐SuppMat.pdf. [file SMTD-10-e70639-s001.pdf]

Supporting information

Structural Profiling of Lipid Nanoparticles at Sub-10 nm Resolution via AF4 Coupled Online to SAXS and SANS

Eva Bittrich, Susanne Boye*, Zanelle Van Niekerk, Zahn Stanvliet, Arthur Porfetye, Fátima Herranz-Trillo, Hans Bolinsson, Stefaniya Gaydarova, Christo Tzachev, Anne Martel, Lars Nilsson, Ralf Schweins, Albena Lederer*

Content

Experimental description of Asymmetrical Flow Field Flow Fractionation (AF4) **p. 2**
Specific considerations for SAXS/SANS detection **p. 4**
Supplemental AF4 data **p. 7**
Supplemental SAXS data **p. 10**
Supplemental SANS data **p. 14**
Tables **p. 17**
References **p. 17**

1. Experimental description of Asymmetrical Flow Field Flow Fractionation (AF4)

All AF4 experiments were performed using an identical channel configuration comprising a trapezoidal short channel (tip-to-tip length 175 mm, maximum width 21 mm) with a 350 µm spacer thickness, fitted with a 10 kDa regenerated cellulose (RC) membrane. To meet the specific requirements and conditions for coupling with SAXS and SANS, and to ensure sufficient signal intensity and quality, different instrumental setups and individually optimized separation flow profiles were employed for the respective systems. For AF4–SAXS (Systems 1 and 2) ultrapure water is used as the carrier because it provides low background in X-ray scattering, is chemically compatible with the samples, and does not introduce additional structural features that would interfere with the SAXS signal. In the case of LNP-p, the recovery was nearly 95%, while LNP-q showed a reduced recovery of ~88% due to the excess quinine, which was partially removed during the focusing step. For AF4-SANS (System 3) D_2O is required instead of H_2O because protiated water yields strong incoherent neutron scattering, whereas D_2O minimizes this background and allows high-quality SANS measurements. In the context of these proof-of-concept studies, recovery tests were not performed because no major differences between H_2O and D_2O in LNP–RC-membrane interactions were expected and resources (time and eluent) were limited.

System 1 (used for AF4-SAXS): AF4 separations were conducted using an Eclipse™ 3+ field-flow fractionation (FFF) (Wyatt Technology, Santa Barbara, CA, USA). Flow control and sample injection were performed using an Agilent 1260 Infinity II pump and autosampler system (Agilent Technologies, USA). The separation system was coupled to multiple detectors: A DAWN™ EOS multi-angle light scattering (MALS) detector (Wyatt Technology, λ = 660 nm) and a diode array UV-Vis detector 1260 (Agilent Technologies, USA), operated at 280 nm relevant to the samples under investigation. The eluent consisted of ultrapure water. Semi-batch measurements were performed by direct channel injection without applied focus and cross-flow at a flow rate of 0.5 mL/min (elution + injection mode). The stock solution of lipid nanoparticles was diluted to a final concentration of 1.04 mg/mL. A total of 0.260 mg of sample was loaded by injecting 250 µL. The following optimized profile was applied for the separation of the samples:

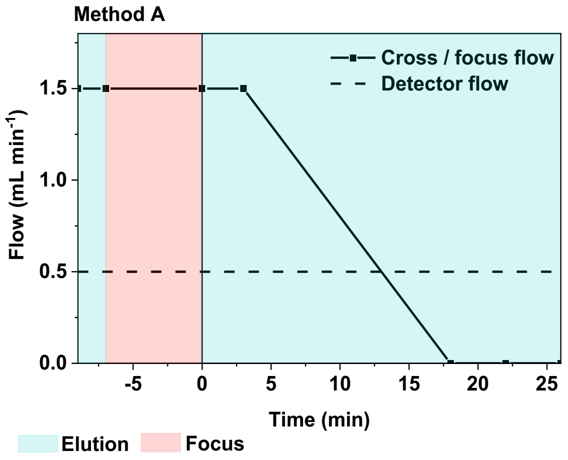

Figure S1. Optimized separation flow profile using AF4 system 1 for AF4-SAXS of solid-lipid nanoparticles.

System 2 (used for AF4-SAXS): AF4 separation was performed using an Eclipse NEON FFF (Waters | Wyatt Technology, USA) with Dilution Control Module (DCM) option coupled to a multi-detector setup consisting of a DAWN NEON multi-angle light scattering (MALS) detector (Waters | Wyatt Technology, USA, λ = 660 nm) with DLS option, an Optilab™ T-rEX™ differential refractive index (dRI) detector (Waters | Wyatt Technology, USA, λ = 660 nm) and UV detector 1260 (Agilent Technologies, USA). Eluent degassing, flow control and sample injection were managed by an Agilent 1260 Infinity II pump and autosampler system (Agilent Technologies, Santa Clara, CA, USA). The split ratio (SR) was set to 8. The eluent consisted of ultrapure water. The stock solution of lipid nanoparticles was diluted to a final concentration of 1.04 mg/mL. A total of 0.260 mg of sample was loaded by injecting 250 µL. The following optimized separation profile was applied:

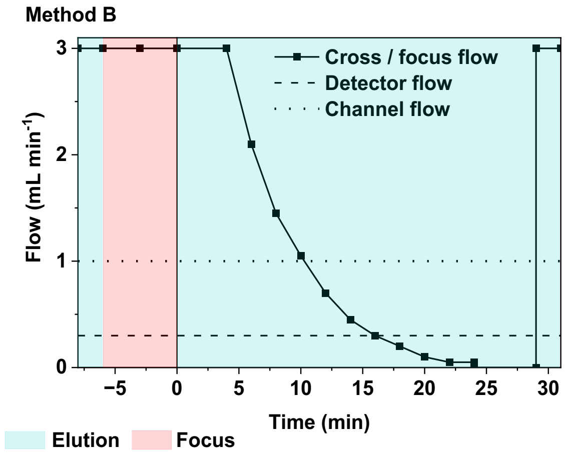

Figure S2. Optimized separation flow profile using AF4 system 2 for AF4-SAXS of solid-lipid nanoparticles.

System 3 (used for AF4-SANS): AF4 separation was performed using an Eclipse NEON FFF (Waters | Wyatt Technology, USA) with Dilution Control Module (DCM) option coupled to a multi-detector setup consisting of a DAWN NEON multi-angle light scattering (MALS) detector (Waters | Wyatt Technology, USA, λ = 658 nm) equipped with wide bore flow cell, attenuators and fluorescence filters (at detectors 4, 6, 8, 10, 12, 14, 16, and 18) and DLS option, an Optilab T-rEX differential refractive index (dRI) detector (Waters | Wyatt Technology, USA, λ = 658 nm) and UV detector SPD-30 (Shimadzu, Japan). Eluent degassing, flow control and sample injection were managed by an Agilent 1260 pump and autosampler system (Agilent Technologies, Santa Clara, CA, USA). The SANS flow cell was placed in between the MALS and the dRI detector. The split ratio (SR) was set to 8. The eluent consisted of D_2O (99.9%) containing 1 mM PBS and 0.9 w/v NaCL. The eluent was filtered prior to use. The stock solution of lipid nanoparticles was diluted to a final concentration of 2.7 mg/mL. A total of 0.405 mg of sample was loaded by injecting 150 µL. The following optimized separation profile was applied:

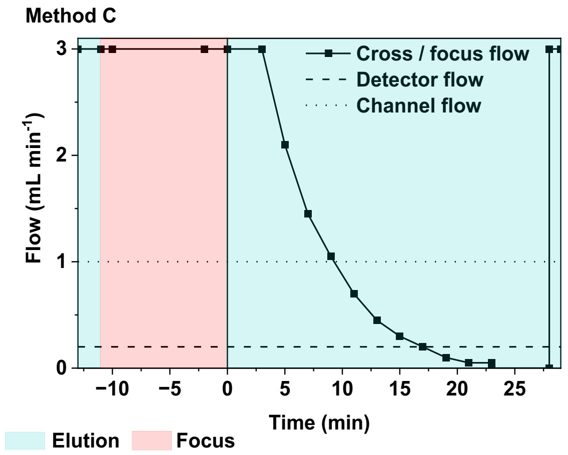

Figure S3. Optimized separation flow profile using AF4 system 3 for AF4-SANS of solid-lipid nanoparticles.

Detailed description of DCM

The dilution control module (DCM)[1] is part of the AF4 channel outlet architecture and flow regulation- It performs a controlled flow split to reduce dilution of the eluting sample. Importantly, the DCM does not pre-concentrate the analyte but limits the intrinsic dilution that occurs when the particle-containing layer mixes with the solvent-rich upper region of the channel. As a result, the analyte reaches the detector at a higher effective concentration, improving the signal-to-noise ratio. The dilution control module (DCM)[1] is positioned directly at the AF4 channel outlet and performs a controlled flow split to reduce dilution of the eluting sample. Importantly, the DCM does not pre-concentrate the analyte but limits the intrinsic dilution that occurs when the particle-containing layer mixes with the solvent-rich upper region of the channel. As

a result, the analyte reaches the detector at a higher effective concentration, improving the signal-to-noise ratio. During AF4 separation, the channel height is typically ~350 µm, whereas eluting species are confined to a thin layer adjacent to the membrane, generally only a few micrometers thick. Consistent with the established concentration profile, the analyte resides almost entirely within ~50 µm of the accumulation wall. Consequently, ~95–99% of the channel cross-section consists of essentially particle-free carrier liquid. When the entire outlet stream is transferred to the detectors, this solvent-rich fraction dilutes the analyte peak.

The DCM reduces this effect by hydraulically partitioning the outlet stream within an integrated outlet section equipped with vertically offset ports. The upper solvent-rich portion is selectively withdrawn, while the particle-enriched lower layer is directed to the detectors. The DCM flow V_{DCM} is defined by the difference between the channel flow V_c and the detector flow V_d:

$V_{DCM} = (V_c - V_d)$

The split ratio (SR) is given by:

$SR = V_{DCM} / V_d$

Thus, increasing the DCM fraction decreases the detector flow and proportionally increases analyte concentration and signal intensity. Because only solvent is removed, the effect is independent of analyte size or physicochemical nature, provided the species remain confined near the membrane during elution. In contrast to increasing injection concentration—which may cause overloading or aggregation—the DCM enhances signal intensity without altering channel conditions, retention behavior, or peak shape, thereby preserving separation performance.

2. Specific considerations for SAXS/SANS detection

Correlation between I(0) and concentration

Online SAXS data were collected without calibration standards, and scattering intensities are therefore used in relative units. SAXS data were corrected for concentration effects by normalization to I_0 as obtained from Guinier analysis, assuming I_0 is only governed by concentration and other influences (particle interaction, aggregation) are absent. In the experimental setup the SAXS cell is positioned downstream of the UV detector and connected by additional tubing. Thus, peak band broadening prevents a direct point-by-point I(0)–UV correlation to prove the hypothesis of sole concentration dependence for I_0. However, the analysis assumes negligible interparticle interactions, supported by previous batch SAXS measurements on LNPs in water, which showed no aggregation and only ~1% change in X-ray transmission between 0.5 and 11 mg/mL. Concentration-dependent absorption was therefore considered not relevant, too. So far the dn/dc or the extinction coefficient of the LNP particles is not known, and no absolute calibration on particle concentration was performed. This would influence fitted scattering length densities (SLDs) in the form factor modeling, but not the extracted sizes.

SANS data were placed on an absolute scale by reference to water. For SANS, I(0) was correlated with an additional UV signal recorded in the SANS cell, giving an approximately linear relationship, which was considered sufficient to justifiy $I_0(c)$. Small deviations occurred for elution times higher than the maximum (35–40 min), but were neglegible. A 0.47 min time lag due to the 30 s SANS frame integration was corrected prior to correlation (see Figure S4).

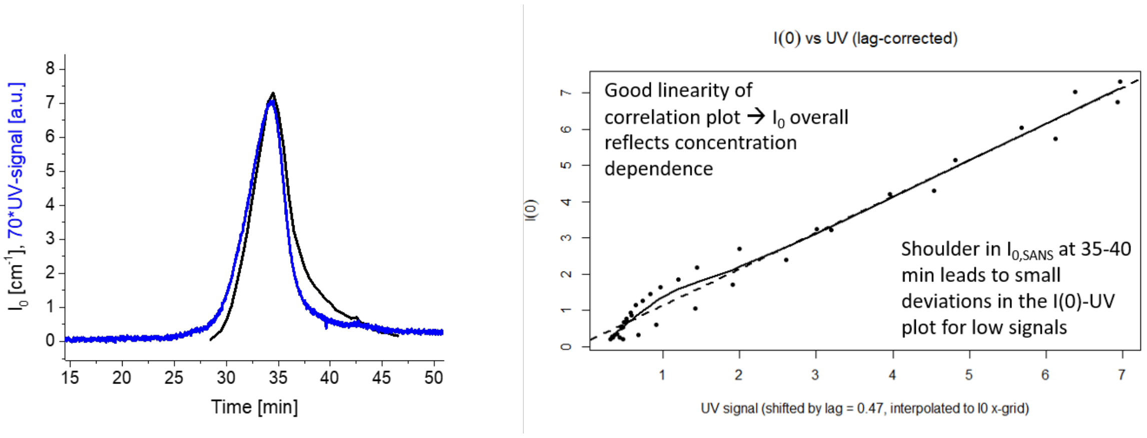

Figure S4. AF4-SANS measurement of **LNP-Q**: Fractograms I0 derived from Guinier analysis of SANS data and UV signal in the SANS flow cell (left), and correlation of SANS and UV intensity (right).

Elliposidal vs Core-shell ellipsoidal form factor models

Form-factor (FF) models were tested in order of increasing complexity: sphere → sphere with polydispersity → core–shell sphere → core–shell sphere with polydispersity → ellipsoid → core–shell ellipsoid. In fact, the polydisperse sphere model can, in principle, reproduce scattering data similar to that of an ellipsoid. For the LNPs, however, cryo-TEM independently confirms a disk-like morphology. More importantly, AF4 separation is used to minimize polydispersity, so that at the minimal frame-integration time of the SAS data the sample can be treated as effectively monodisperse. We therefore regard the ellipsoidal FF as highly appropriate for describing the LNP scattering data, and apply the core–shell ellipsoid FF wherever the contrast allows.

Ellipsoidal and core–shell ellipsoidal FF models as implemented in SasView were used for data analysis.

Model documentation available in the SasView online manual:
https://www.sasview.org/docs/user/models/ellipsoid.html and
https://www.sasview.org/docs/user/models/core_shell_ellipsoid.html.

For the SANS data, instrumental smearing was included via dQ and weighting via dI data; no additional restraints were applied and the full range $0.0062 < q < 0.65$ Å^{-1} was fitted.

Both models gave stable fits for the SANS data in Fig. 3c with $\chi^2 = 7.5$ (ellipsoid) and reduced $\chi^2 = 3.6$ (core–shell). The ellipsoid model uses four free parameters, the core–shell ellipsoid seven; starting values for the core–shell fits were taken from the ellipsoid results, and varying these starting values always converged to the same solution for LNP-Q SANS data. Because of its lower χ^2, the core–shell ellipsoid model was selected, and the final fit parameters with uncertainties are listed in Table S1.

Precise calculation of the core-shell SLD contrast is feasible, because even small changes in SLD produce simulated scattering curves that exceed the experimental noise. We therefore consider the contrast sufficient to justify discussing distinct core and shell contributions and to enable comparisons between samples (e.g., LNP-p vs LNP-Q).

Table S1. Fit parameters, values and errors for ellipsoidal and core-shel FF models of LNP-Q SANS data.

**Fit parameter**	**Fitted value**	**Fit error**
Ellipsoid FF		
Background	$2.59E^{-5}$	$1.8E^{-6}$
SLD	6.357 10^{-6}/Å^2	7.7E-5 10^{-6}/Å^2
Radius_polar	77.2 Å	0.4 Å
Radius_equatorial	183.3 Å	1.4 Å
Core-shell ellipsoid FF		
Background	$2.59E^{-5}$	$1.8E^{-6}$
Radius_equat_core	125.0	4.5
X_core	0.40	0.04
Thick_shell	57 Å	4 Å
X_polar_shell	0.47	0.09
SLD_core	6.3512 10^{-6}/Å2	0.0004 10^{-6}/Å^2
SLD_shell	6.3591 10^{-6}/Å2	0.0006 10^{-6}/Å^2

3. Supplemental AF4 data

Fractograms AF4-MALS

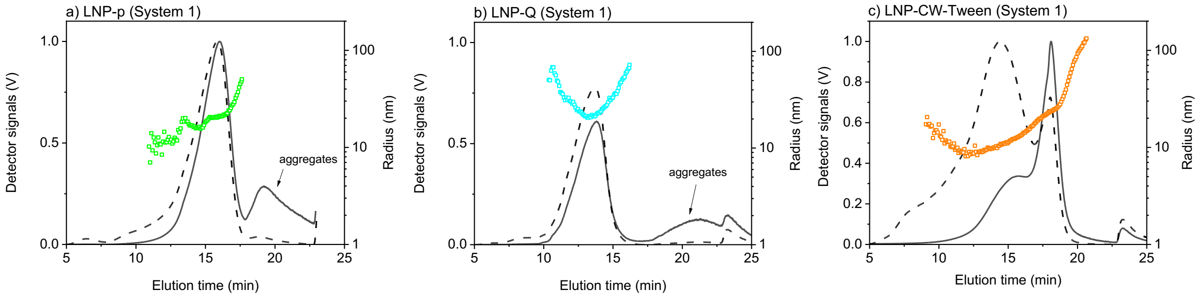

Figure S5. Fractograms of a) LNP-p; b) LNP-Q and c) LNP-CW-Tween; detector signals (solid line- normalized MALS (90°) and dashed line – UV $_{280nm}$) and radii (square – R_g) vs elution time (starting after focusing) using AF4 system 1 for SAXS coupling.

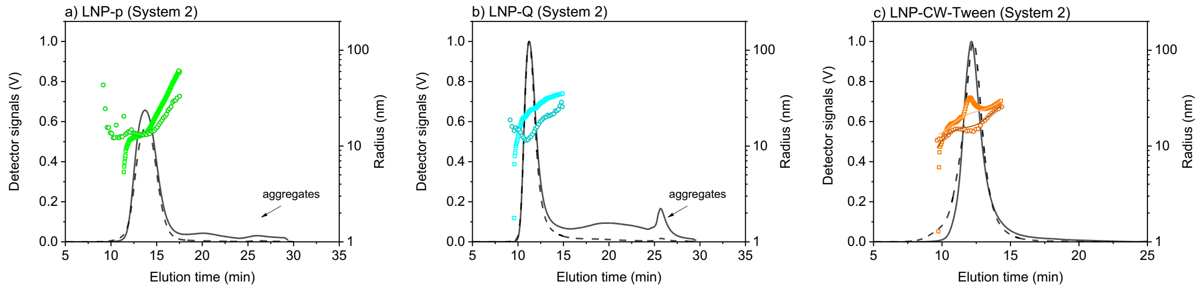

Figure S6. Fractograms of a) LNP-p; b) LNP-Q and c) LNP-CW-Tween; detector signals (solid line- MALS (90°) and dashed line – UV $_{280nm}$) and radii (square – R_g; circle – R_h) vs elution time (starting after focusing) using AF4 system 2 for SAXS coupling.

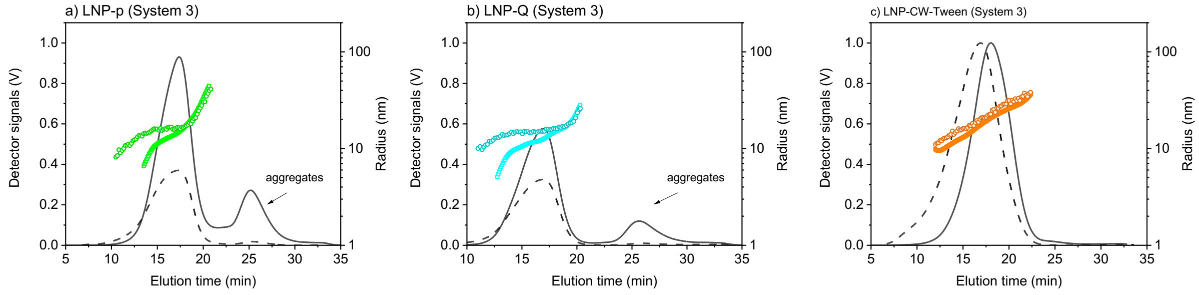

Figure S7. Fractograms of a) LNP-p; b) LNP-Q and c) LNP-CW-Tween; detector signals (solid line- MALS (90°) and dashed line – UV $_{280nm}$) and radii (square – R_g; circle – R_h) vs elution time (starting after focusing) using AF4 system 3 for SANS coupling.

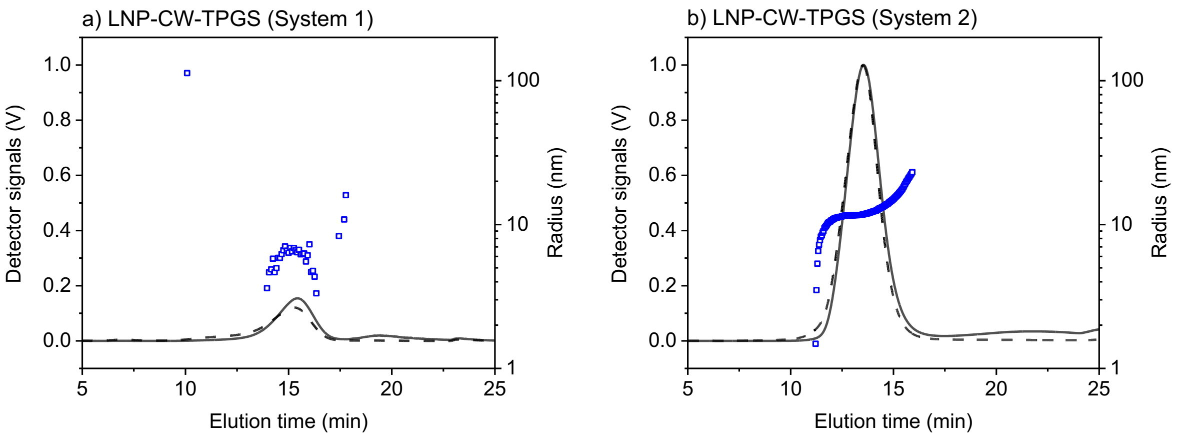

Figure S8. Fractograms of LNP-CW-TPGS using a) AF4 system 1 and b) AF4 system 2; detector signals (solid line- MALS (90°) and dashed line – UV $_{280nm}$) and radii (square – R_g) vs elution time (starting after focusing) using AF4 - SAXS coupling.

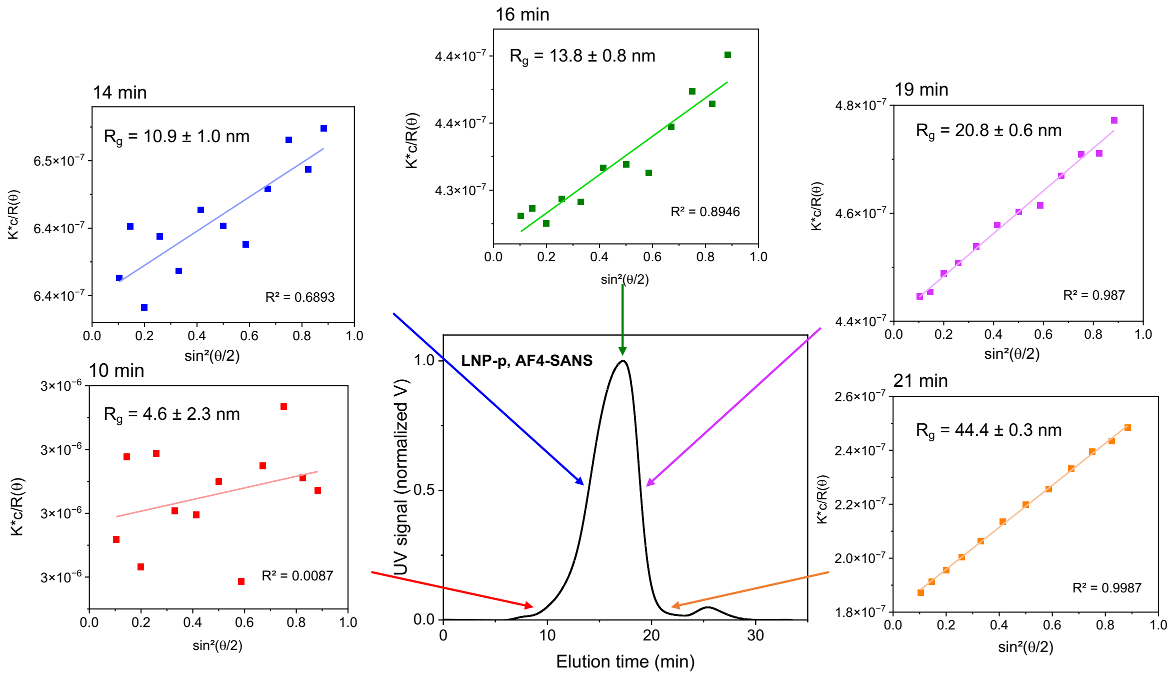

Figure S9. Zimm plots of MALS data at different elution times (obtained by AF4 system 3) of LNP-p based on UV fractogram.

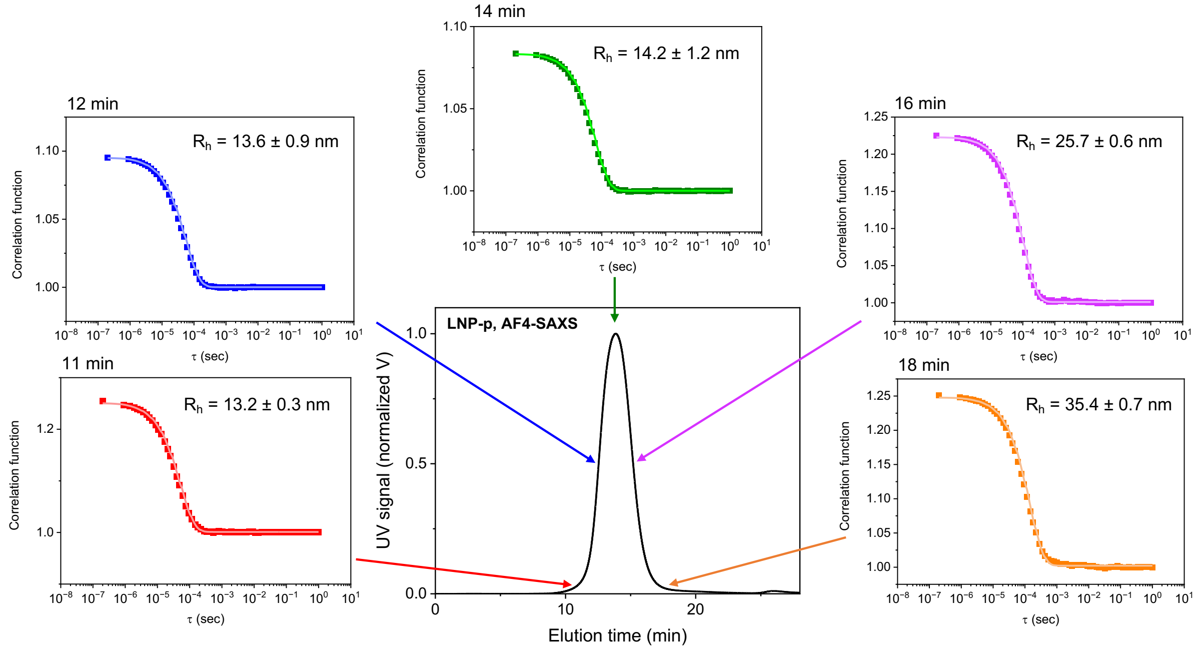

Figure S10. Correlation curves as derived from DLS data beyond the elution maximum of LNP-p, using AF4 system 2.

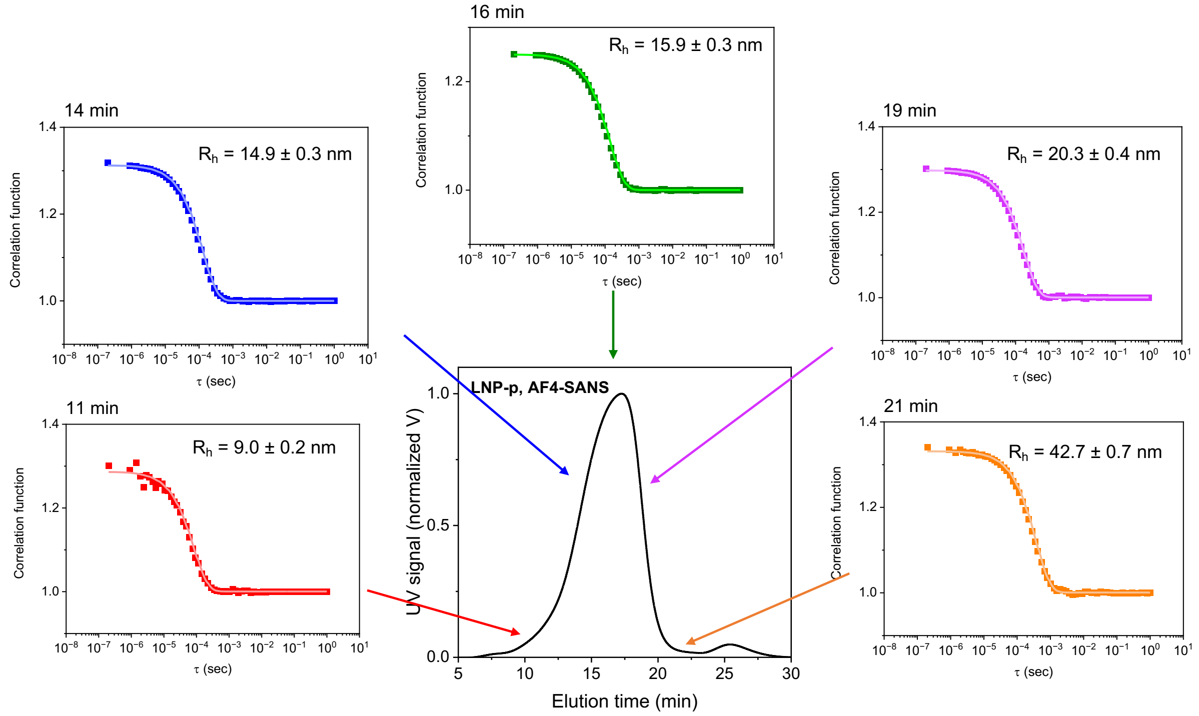

Figure S11. Correlation curves as derived from DLS data beyond the elution maximum of LNP-p, using AF4 system 3.

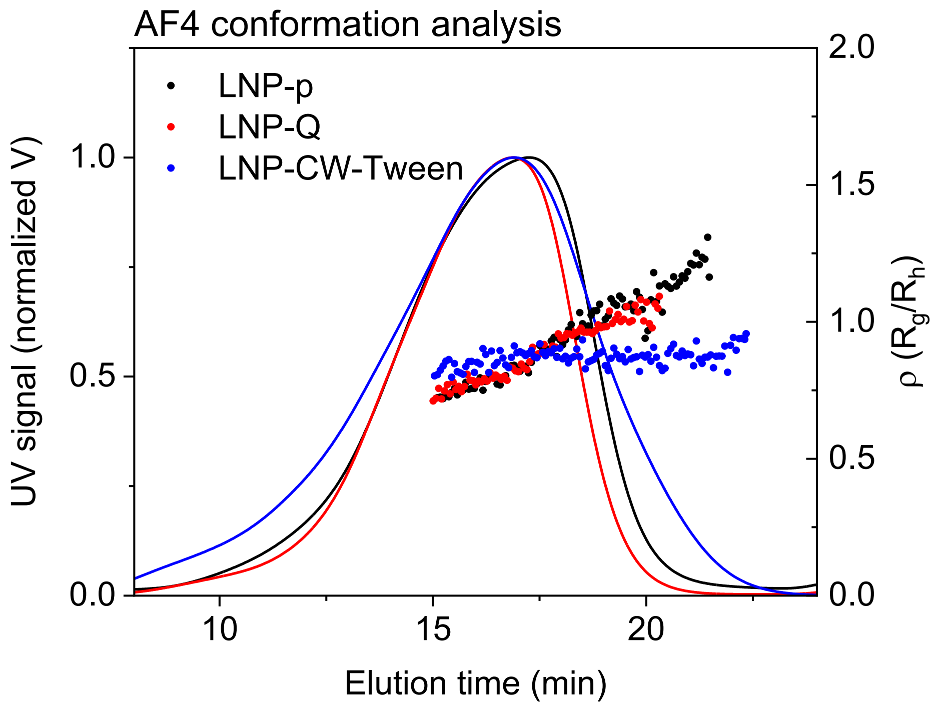

Figure S12. Burchard-Stockmayer plot: normalized UV signals (lines) and ρ (R_g/R_h) (symbols) of LNP-p (black); LNP-Q (red) and LNP-CW-Tween (blue) vs. elution time (starting after focusing) using AF4 system 3 for SANS coupling. The shape parameter ρ is theoretically calculated with 0.65 to 1.5 for ellipsoidal particles depending on the shape (prolate or oblate) which is in agreement wth the obesreved values in this plot (0.65 to 1.3).

4. Supplemental SAXS data

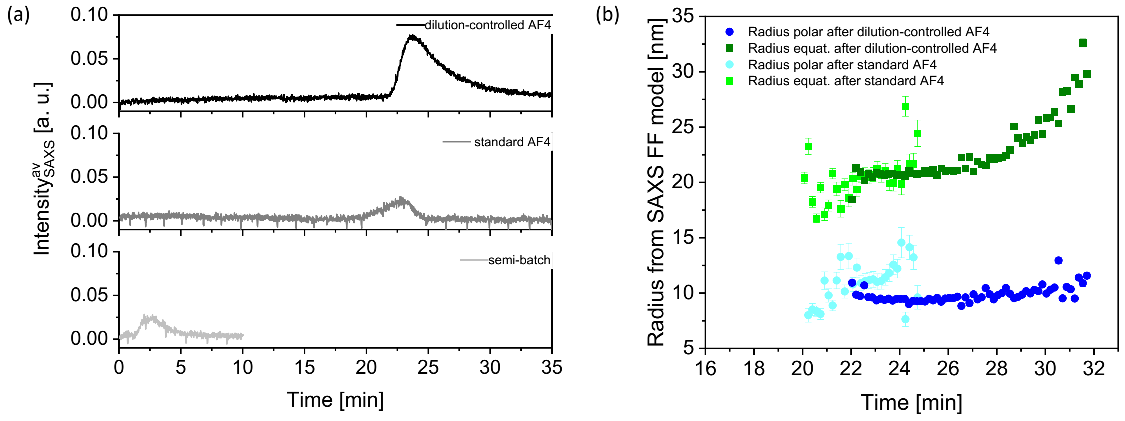

Figure S13. (a) Average SAXS data over time and (b) polar and equatorial radii of the elliptical LNP-Q obtain by form factor modeling. AF4 separation and SAXS measurements were started in parallel. Displayed SAXS data is not corrected for the focusing time of AF4.

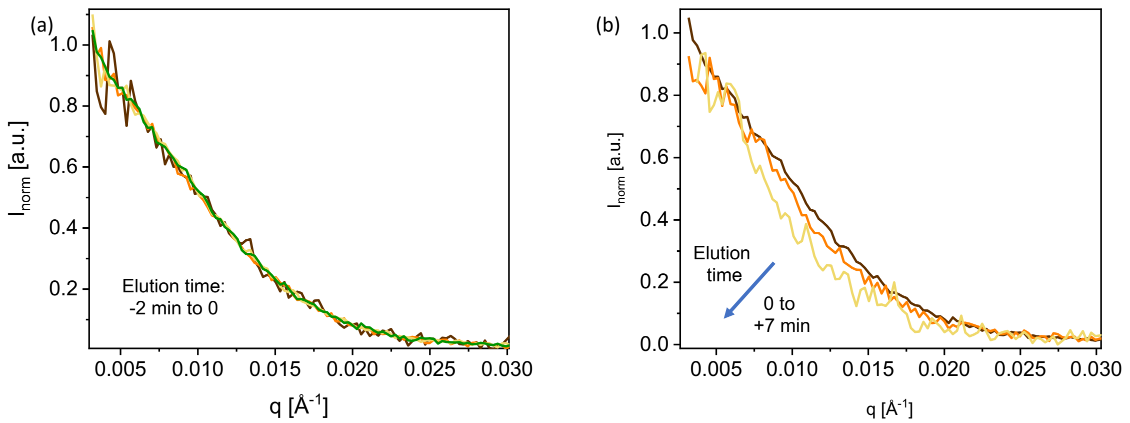

Figure S14. Normalized SAXS data of LNP-Q for different time points. Normalization was performed to I_0 (Fig. S16) as obtained from Guinier fits (Fig. S15) to correct for the concentration dependence in the data.

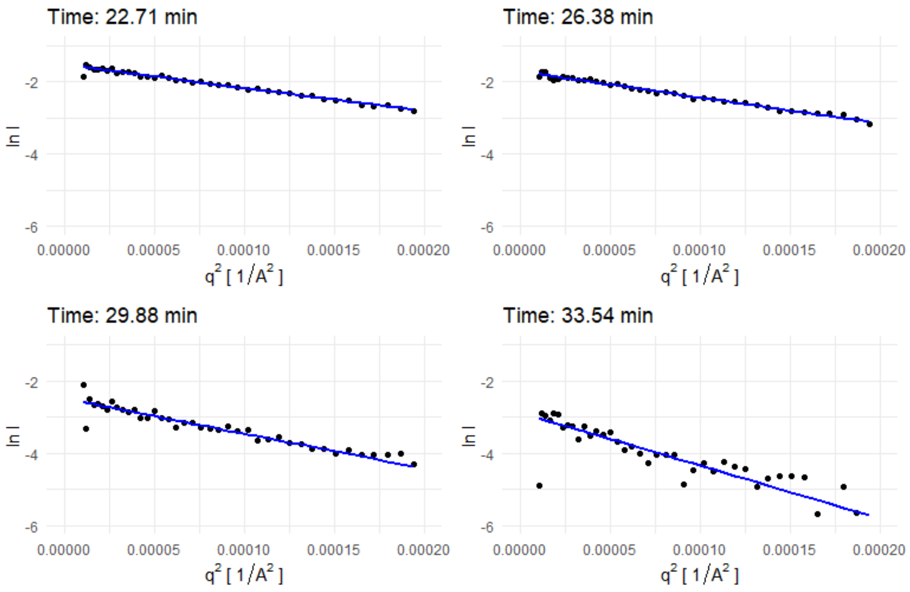

Figure S15. Examples of Guinier fits of SAXS data of LNP-Q for different time points of absolute SAXS measurement time.

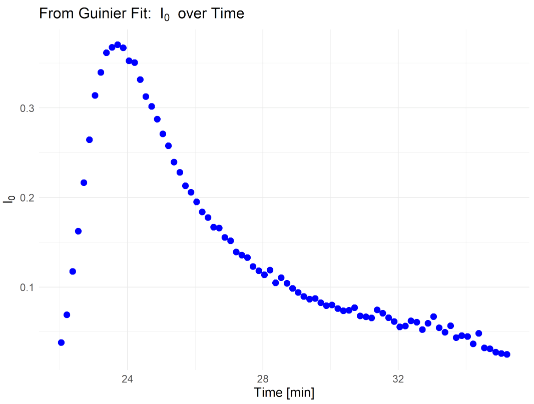

Figure S16. Intensity I_0 [a. u.] for LNP-Q with time as derived from Guinier fits of SAXS data.

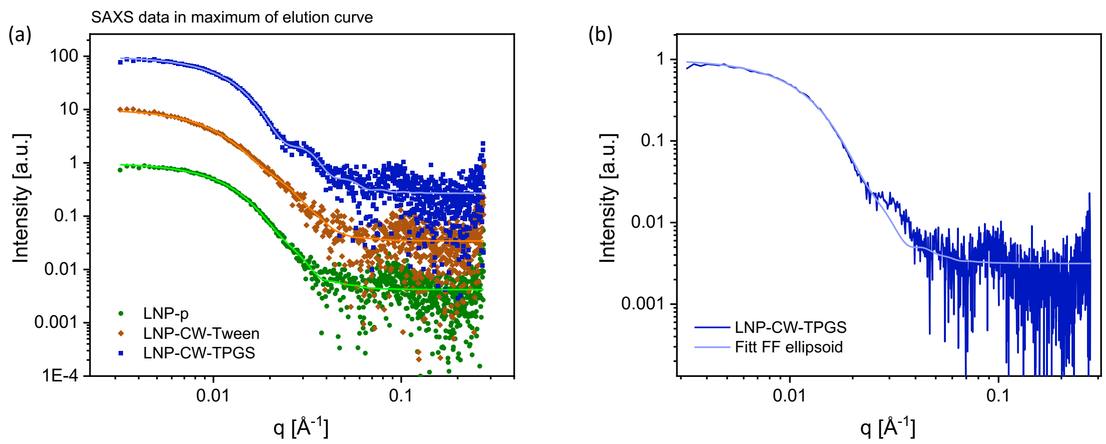

Figure S17. (a) SAXS data in the maximum of the elution curve for LNP-p, LNP-CW-Tween, and LNP-CW-TPGS after dilution-controlled AF4 with corresponding fit functions after FF modeling (ellipsoids of revolution for LNP-p and LNP-CW-Tween, core-shell ellipsoid for LNP-CW-TPGS). The data is shifted by a factor of 10 (LNP-CW-Tween) and 100 (LNP-CW-TPGS) in intensity for clear display. (b) Test FF model of ellipsoid of revolution for LNP-CW-TPGS.

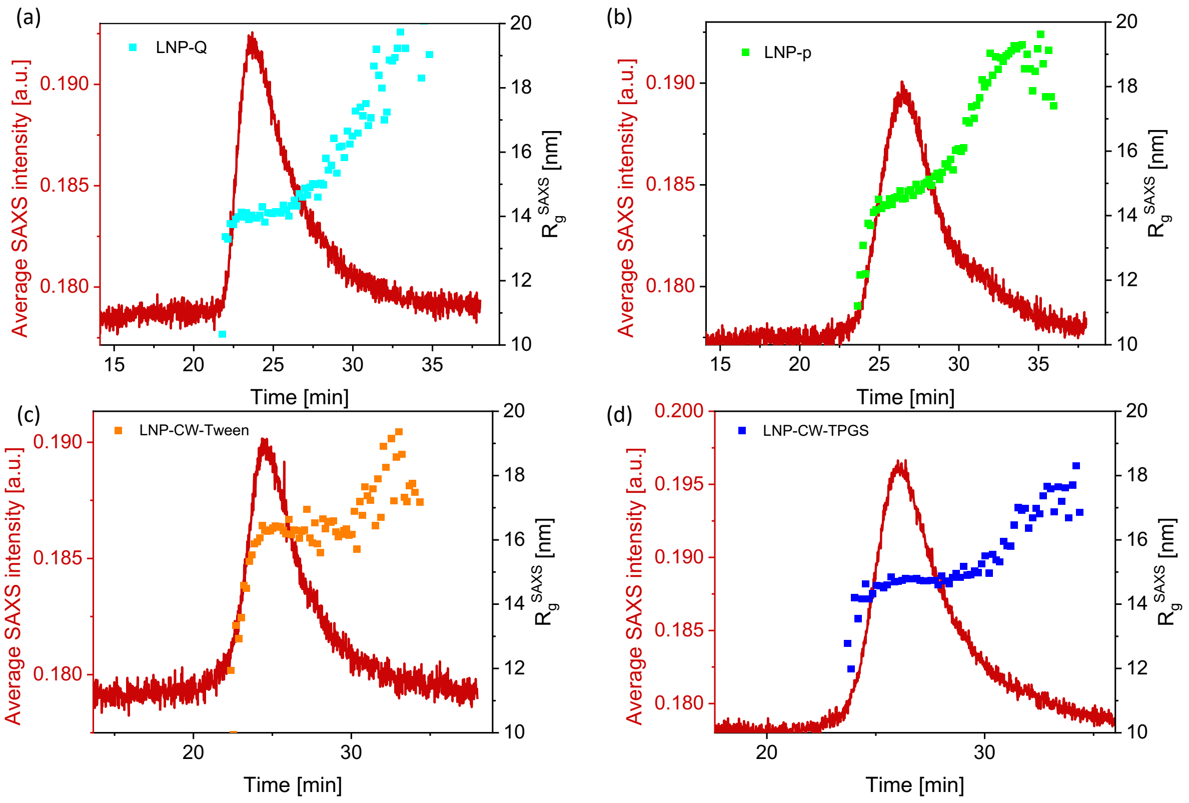

Figure S18. Average SAXS intensity over absolute measurement time and radius of gyration R_g as derived by Guinier analysis of time-dependent SAXS data for LNP-Q (a), LNP-p (b), LNP-CW-Tween (c), and LNP-CW-TPGS (d). Time axis is not corrected for the focusing time of the AF4 separation.

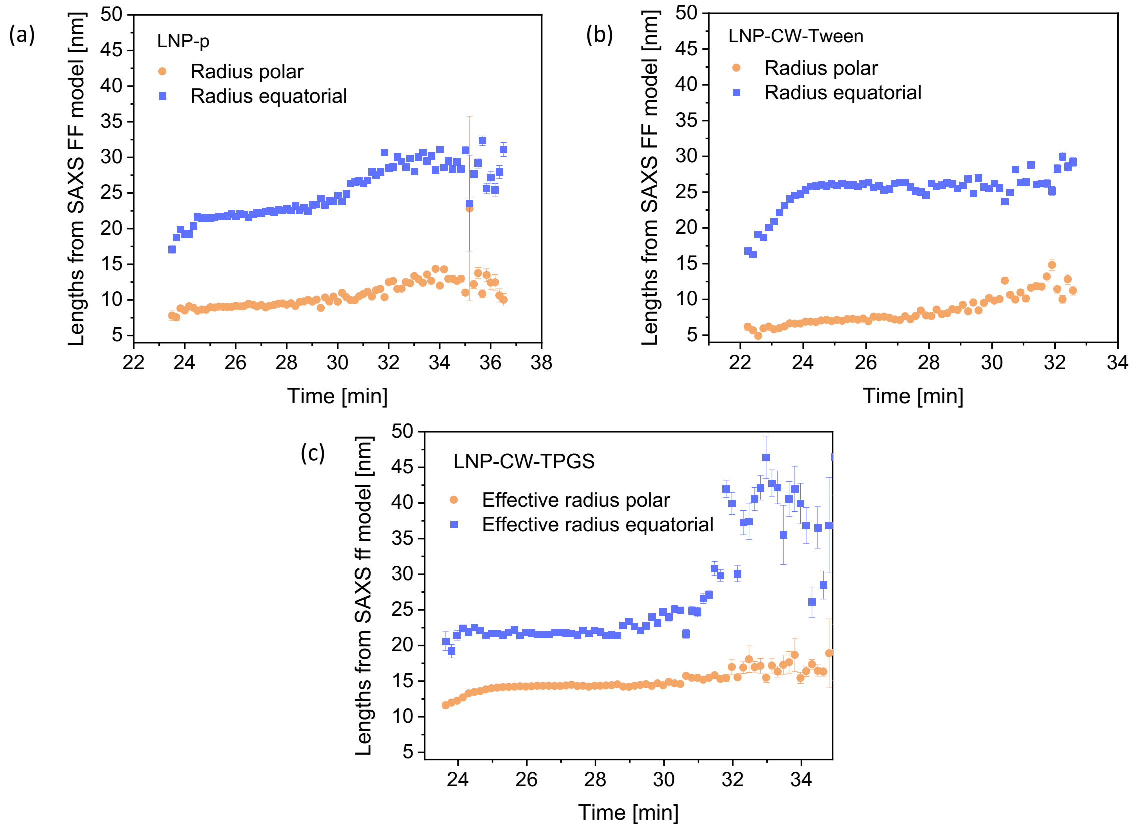

Figure S19. Polar and equatorial radii dependent on absolute time of the elliptical LNP-p (a), LNP-CW-Tween (b), and LNP-CW-TPGS (c) obtain by form factor modeling of SAXS data. For LNP-CW-TPGS radii were calculated from a core and shell radii of a core-shell ellipsoid FF model. For the radii of LNP-Q in absolute time scale see Fig. S13. Time is not corrected for the focusing time of the AF4 separation.

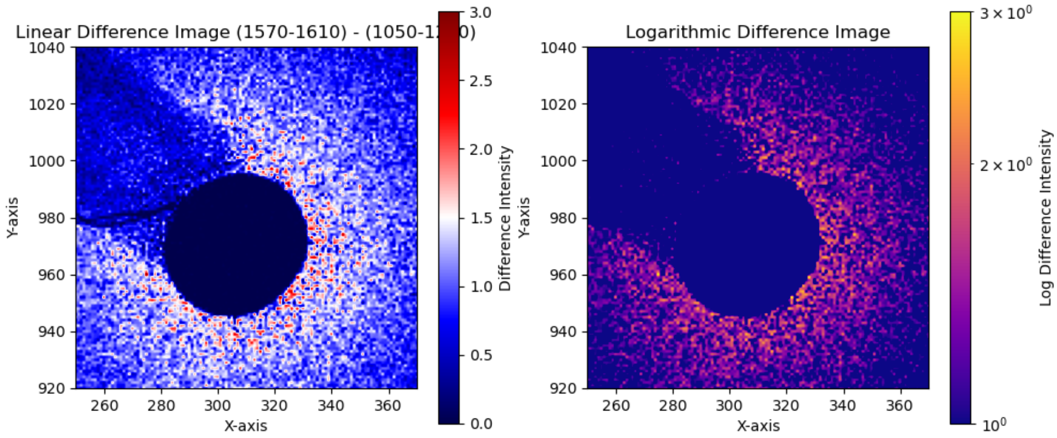

Figure S20. 2D detector images of background corrected SAXS data of LNP-p in the maximum of elution, integrated for 40 s in linear intensity scale (a) and in logarithmic scale (b). No anisotropic scattering signal of oriented particles is observed.

5. Supplemental SANS data

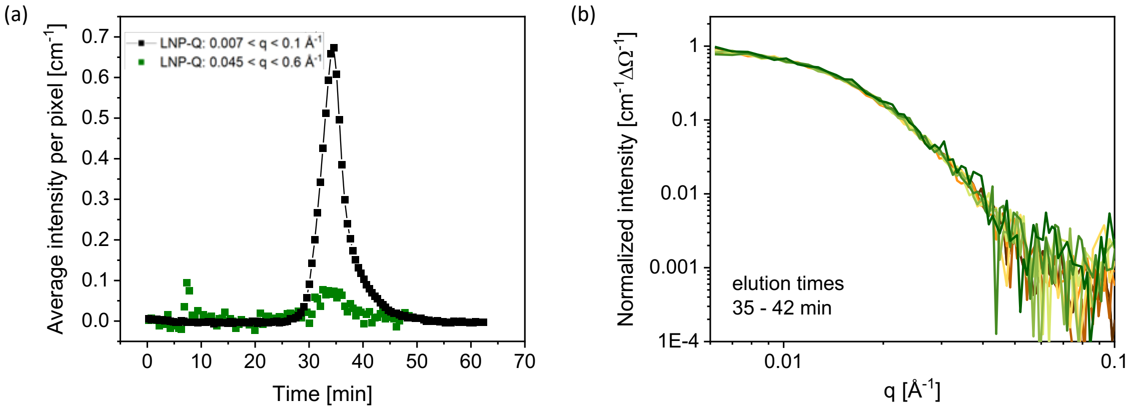

Figure S21. (a) Averaged SANS intensity versus absolute time for LNP-Q at two different detector distances (10.2 m and 1.6 m). (b) SANS data for the LNP-Q versus q for elution times higher than at the maximum of elution. Data is normalized to I_0 (Fig. S23) as obtained from Guinier fits (Fig. S22).

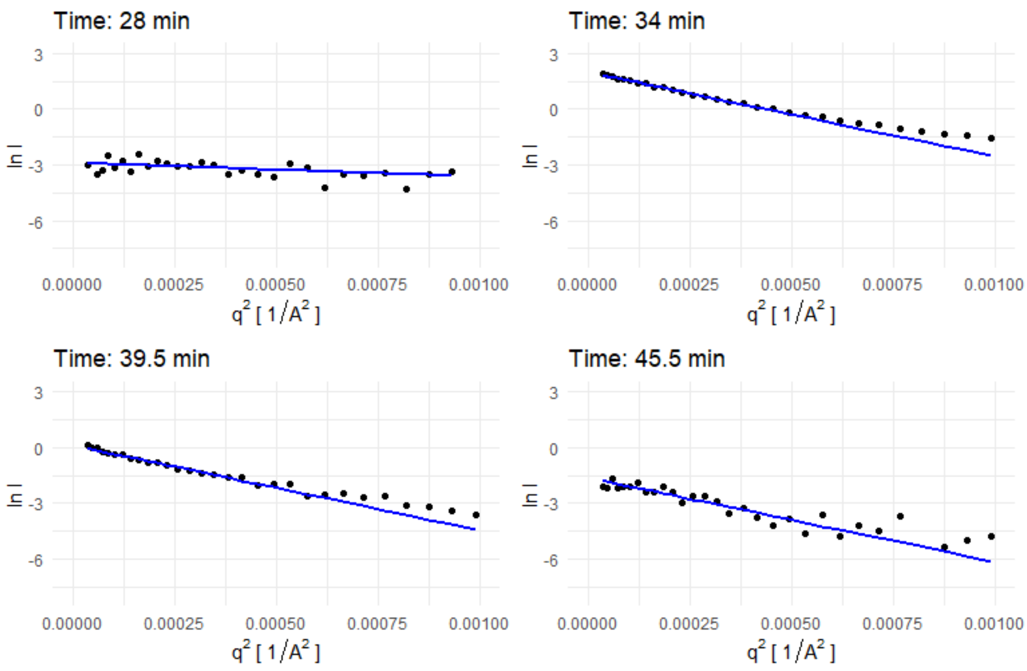

Figure S22. Examples of Guinier fits of SANS data of LNP-Q with time.

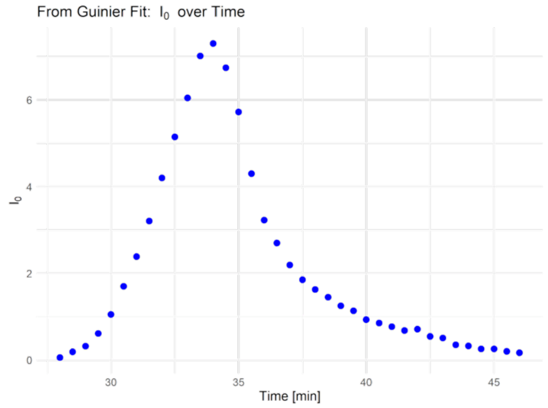

Figure S23. Intensity I_0 [cm^{-1}] for LNP-Q with time as derived from Guinier fits of SANS data.

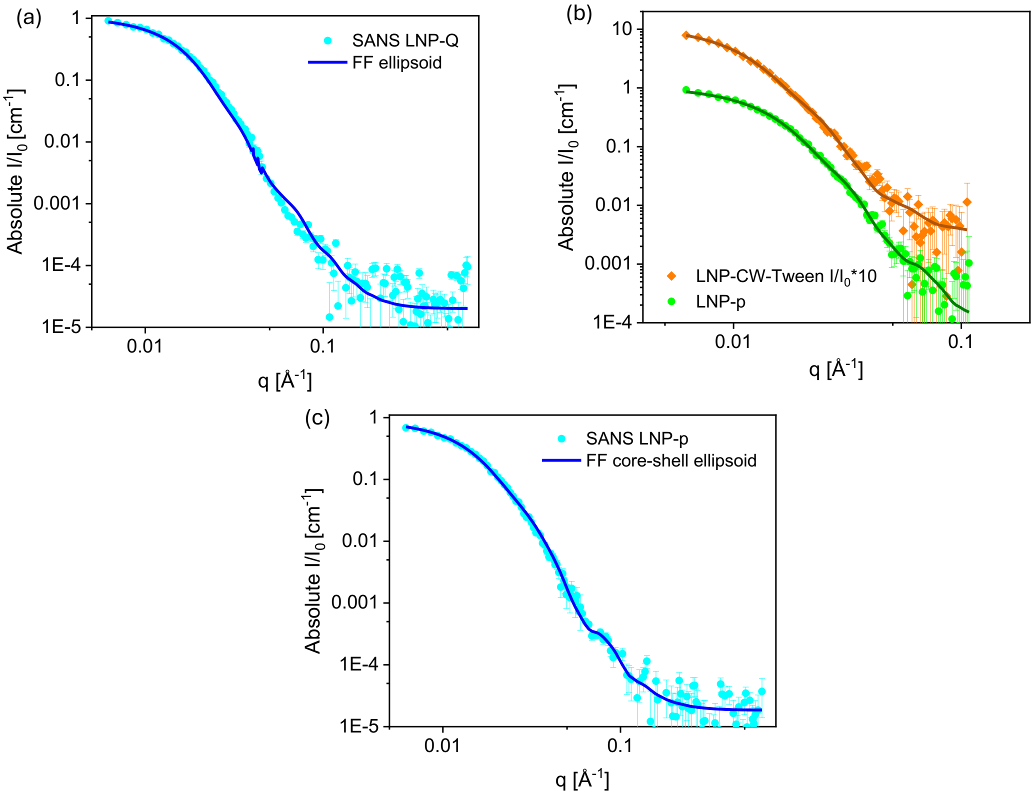

Figure S24. (a) Test of ellipsoidal form factor model for SANS data of LNP-Q. (b) SANS data at 10 m detector distance in the elution maximum (30 s frame) for LNP-p and LNP-CW-Tween, and ellipsoidal FF models. (c) Core-shell ellipsoidal form factor model for SANS data of LNP-p, with fitted SLDs of $(6.352 \pm 0.001) \cdot 10^{-6}$ Å^{-2} and $(6.365 \pm 0.001) \cdot 10^{-6}$ Å^{-2} for the shell.

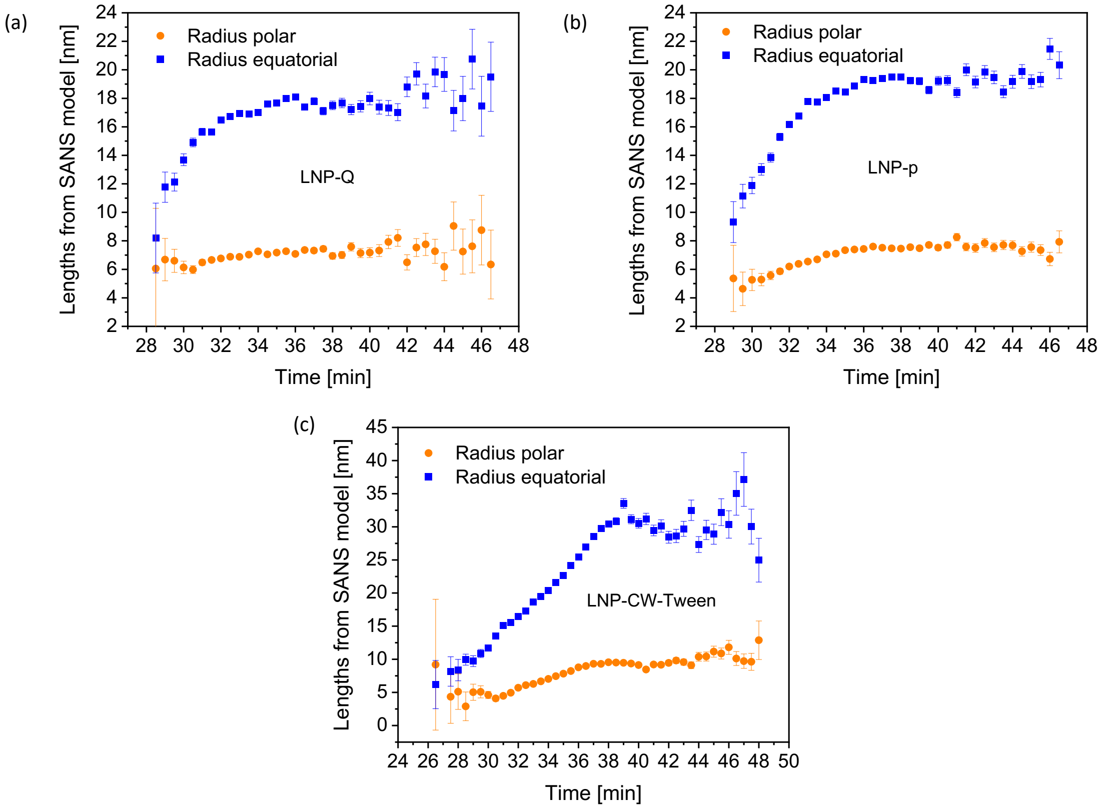

Figure S25. Average SANS intensity after dilution-controlled AF4 and corresponding evaluated Rg values with time for LNP-Q (a), LNP-p (b), and LNP-CW-Tween (c).

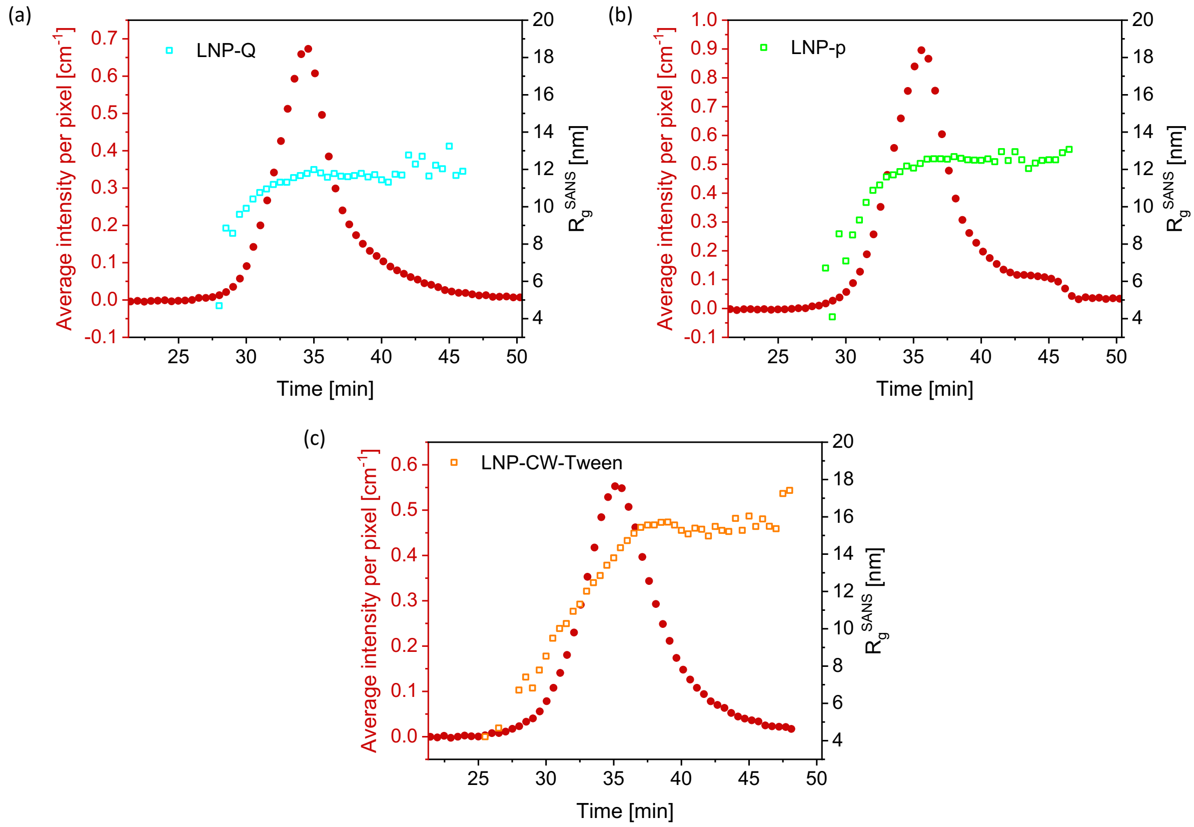

Figure S26. Average SANS intensity after dilution-controlled AF4 and corresponding evaluated R_g values with time for LNP-Q (a), LNP-p (b), and LNP-CW-Tween (c).

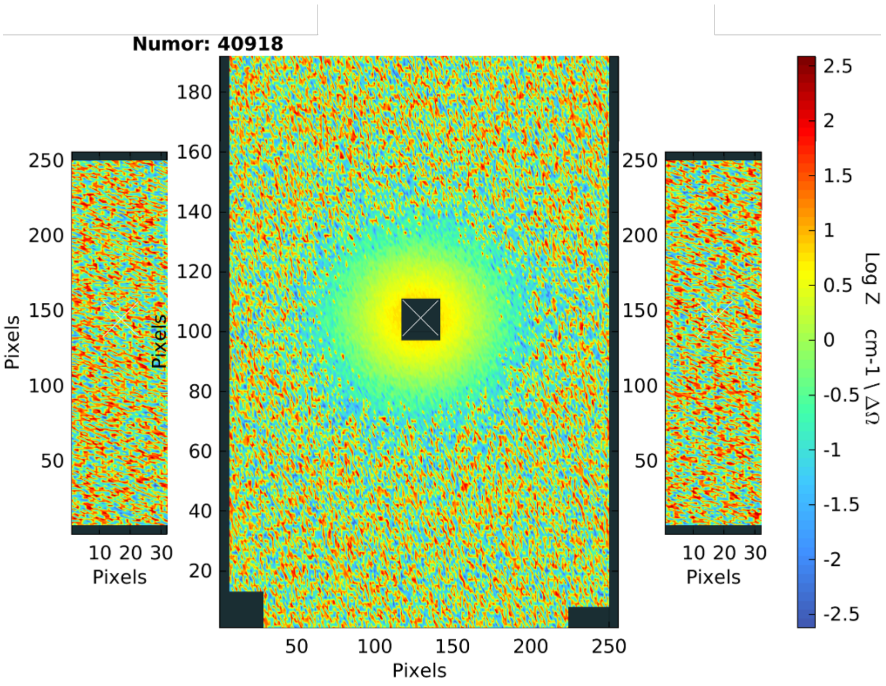

Figure S27. 2D detector image of background corrected absolute intensity data for LNP-p in the maximum of the elution curve integrated for 2 min. No anisotropic scattering pattern indicative or oriented particles in the flow could be found.

6. Tables

Table S2. Results from AF4 coupled to MALS and DLS measurements of different LNPs using system 1. Note that R_g from MALS is interpreted in the range >15 nm due to the limits of the light scattering detection at λ = 660 nm.

Sample	$R_{g, MALS}$ (nm)	$R_{h, DLS}$ (nm)
LNP-p	20.3 ± 2.6	n.a.
LNP-Q	28.4 ± 2.1	n.a.
LNP-CW-Tween	18.0 ± 1.1	n.a.

Table S3. Results of AF4 measurements of different LNPs using system 2. Note that R_g from MALS is interpreted in the range >15 nm due to the limits of the light scattering detection at λ = 660 nm.

Sample	$R_{g, MALS}$ (nm)	$R_{h, DLS}$ (nm)
LNP-p	19.6 ± 1.0	14.6 ± 1.4
LNP-Q	23.5 ± 1.0	13.7 ± 1.9
LNP-CW-Tween	23.4 ± 0.2	16.7 ± 0.5

Table S4. Results of AF4 measurements of different LNPs using system 3. Note that R_g from MALS is interpreted in the range >15 nm due to the limits of the light scattering detection at λ = 660 nm.

Sample	$R_{g, MALS}$ (nm)	$R_{h, DLS}$ (nm)	ρ (R_g/R_h) at concentration maximum
LNP-p	18.7 ± 0.1	16.5 ± 0.4	0.85 ± 0.02
LNP-Q	20.1 ± 1.2	15.0 ± 0.4	0.83 ± 0.02
LNP-CW-Tween	21.9 ± 1.3	20.1 ± 0.4	0.88 ± 0.02

Table S5. Calculated scattering length densities for the components in the LNP. All data was obtained with a density of 1 g/cm^3, for D_2O a density of 1.11 g/cm^3 was used.

Component	Sum formula	SLD [10^{-6}/Å^2] X-ray: 0.99 Å	Real SLD [10^{-6}/ Å^2] Neutrons: 5.24 Å
Carnauba wax	mostly aliphatic esters	8.7 … 9.6	-1 … 2
Red palm oil:			
42% Palmitic acid	C16H32O2	9.541	-0.041
42% Oleic acid	C18H34O2	9.504	0.087
Polysorbat 40	C62H122O26	9.295	0.500 (in H2O)<br>0.555 (in D2O)
D-α-tocopherol-polyethylenglycol-1000-succinate (TPGS)	C35H58O6(C2H4O)23	9.290	0.522 (in H2O)<br>0.613 (in D2O)
	H2O	9.442	-0.561
	D2O		6.388
Quinine	C20H24N2O2		1.364

7. References:

[1] Johann C, Deng C. WP2900: Innovations in FFF-Eclipse NEON n.d.:1–9. https://wyattfiles.s3-us-west-2.amazonaws.com/literature/white-papers/WP2900-Innovations-in-FFF-MALS-DLS-Eclipse-NEON.pdf
